# Nuclear Fructose‐1,6‐Bisphosphate Inhibits Tumor Growth and Sensitizes Chemotherapy by Targeting HMGB1

**DOI:** 10.1002/advs.202203528

**Published:** 2023-01-15

**Authors:** Yeyi Li, Yuan Fu, Yan Zhang, Bilian Duan, Yanli Zhao, Man Shang, Ying Cheng, Kai Zhang, Qiujing Yu, Ting Wang

**Affiliations:** ^1^ National Clinical Research Center for Cancer Key Laboratory of Cancer Prevention and Therapy Tianjin's Clinical Research Center for Cancer Tianjin Lung Cancer Center Department of Thoracic Oncology Tianjin Cancer Institute and Hospital Tianjin Key Laboratory of Inflammatory Biology The Province and Ministry Co‐sponsored Collaborative Innovation Center for Medical Epigenetics Department of Pharmacology School of Basic Medical Sciences Tianjin Medical University Cancer Institute and Hospital Tianjin Medical University Tianjin 300060 China; ^2^ Center for Mitochondrial Biology & Medicine the Key Laboratory of Biomedical Information Engineering of Ministry of Education School of Life Science and Technology Xi'an Jiaotong University Xi'an 710049 China; ^3^ The Province and Ministry Co‐sponsored Collaborative Innovation Center for Medical Epigenetics Tianjin Key Laboratory of Medical Epigenetics Key Laboratory of Immune Microenvironment and Disease (Ministry of Education) Department of Biochemistry and Molecular Biology Tianjin Medical University Tianjin 300070 China; ^4^ Key Laboratory of Immune Microenvironment and Disease (Ministry of Education) Department of Immunology School of Basic Medical Sciences Tianjin Medical University Tianjin 300070 China

**Keywords:** cancer, chemotherapy, DNA repair, HMGB1, P53

## Abstract

Metabolites are important for cell fate determination. Fructose‐1,6‐bisphosphate (F1,6P) is the rate‐limiting product in glycolysis and the rate‐limiting substrate in gluconeogenesis. Here, it is discovered that the nuclear‐accumulated F1,6P impairs cancer cell viability by directly binding to high mobility group box 1 (HMGB1), the most abundant non‐histone chromosome structural protein with paradoxical roles in tumor development. F1,6P disrupts the association between the HMGB1 A‐box and C‐tail by targeting K43/K44 residues, inhibits HMGB1 oligomerization, and stabilizes P53 protein by increasing P53–HMGB1 interaction. Moreover, F1,6P lowers the affinity of HMGB1 for DNA and DNA adducts, which sensitizes cancer cells to chemotherapeutic drug(s)‐induced DNA replication stress and DNA damage. Concordantly, F1,6P resensitizes cancer cells with chemotherapy resistance, impairs tumor growth and enhances chemosensitivity in mice, and impedes the growth of human tumor organoids. These findings reveal a novel role for nuclear‐accumulated F1,6P and underscore the potential utility of F1,6P as a drug for cancer therapy.

## Graphical abstract

F1,6P is an inter‐metabolite in glycolysis as well as a clinically used drug. However, in cancer cells with F1,6P treatment, nuclear‐accumulated F1,6P directly targets to HMGB1, which promotes the anti‐tumor but inhibits the pro‐tumor functions of HMGB1, suggesting a potential role for F1,6P as an HMGB1‐targeting drug for cancer therapy, alone or in combination with chemotherapy.

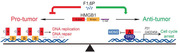

## 1 Introduction

Cell metabolism refers to the sum of chemical reactions (usually small molecular) in living cells. Since the discovery of the *lac* operon^[^ ^1^ ^]^ in 1961, it is well known that these chemicals, also named metabolites, regulate cellular activities in both metabolic reaction‐dependent and ‐independent ways, in which metabolites also act as natural ligands to regulate the functions of various proteins.

F1,6P is generated at the rate‐limiting step of glycolysis by phosphofructokinase.^[^ ^2^ ^]^ Therefore, intracellular F1,6P is sustained but restrained at a certain level that is determined by glucose availability. On the other hand, F1,6P is also the substrate of fructose‐1,6‐bisphosphatase (FBP1), the rate‐limiting enzyme in gluconeogenesis, which maintains the continuous supply of blood glucose.^[^ ^3^ ^]^ In addition, F1,6P has also been reported as a regulatory ligand for proteins other than metabolic enzymes, such as the signaling protein Sos1/Ras^[^ ^4^ ^]^ in yeast and the transcription factors CggR^[^ ^5^ ^]^ and CcpA^[^ ^6^ ^]^ in bacteria. The interaction between F1,6P and these non‐metabolic proteins integrates nutrient availability with intracellular signals to coordinate decisions in cell fate and nutrient utilization in these single‐celled organisms.

Moreover, exogenous F1,6P can be directly transported across the cell membrane^[^ ^7^ ^]^ and displays a cytoprotective function in cultured cardiomyocytes and neurons.^[^ ^8^ ^]^ The administration of F1,6P in vivo shows cardioprotective effects against ischemic damage in mice^[^ ^9^ ^]^ as well as in human clinical trials.^[^ ^10^ ^]^ Therefore, F1,6P has been used clinically as an adjuvant drug for anoxia‐ or ischemia‐related heart and brain disease in several countries including China.

HMGB1 is an essential gene for blood glucose homeostasis, and HMGB1 knockout (KO) causes lethal hypoglycemia in mice.^[^ ^11^ ^]^ HMGB1 protein was first isolated from rat liver and was found to bind preferentially to single‐strand DNA (ssDNA).^[^ ^12^ ^]^ The purified protein exists as an oligomer both in physiological buffer and when complexed with DNA.^[^ ^13^ ^]^ As a non‐histone chromosome structural protein, HMGB1 is functionally involved in various DNA‐associated processes, such as transcriptional regulation of genes related to gluconeogenesis.^[^ ^11^ ^]^ However, HMGB1 binds to DNA in a non‐specific manner and its gene specificity still cannot be readily explained.^[^ ^14^, ^15^ ^]^ In contrast, since HMGB1 binds preferentially to ssDNA or to various types of DNA adducts,^[^ ^16^, ^17^, ^18^, ^19^ ^]^ its functions in DNA replication and DNA repair have been well characterized. Additionally, the roles of HMGB1 as a P53 chaperone^[^ ^20^ ^]^ as well as a secreted inflammatory cytokine^[^ ^21^ ^]^ have also been extensively studied.

HMGB1 is often up‐regulated in human cancers. However, as a multifunctional protein, HMGB1 displays paradoxical roles in cancer development or cancer therapy, promoting both cancer cell survival and death through complicated mechanisms.^[^ ^22^ ^]^ Thus, developing HMGB1‐targeting agents for cancer therapy is challenging.

In this study, we found that nuclear F1,6P impairs cancer cell viability and sensitizes chemotherapy. Mechanistically, F1,6P directly binds to and deoligomerizes HMGB1 protein, which leads to increased HMGB1–P53 association as well as decreased HMGB1–DNA affinity. As a result, F1,6P suppresses cancer cell proliferation through P53 and aggravates chemotherapeutic drug‐induced cell death by impairing the DNA‐associated functions of HMGB1 like DNA replication and repair. Thus, F1,6P has been identified as a novel HMGB1 ligand and potentially an HMGB1‐targeting drug for cancer therapy.

## 2 Results and Discussion

### 2.1 Nuclear Accumulation of F1,6P Impairs Cancer Cell Viability

**Figure 1:**
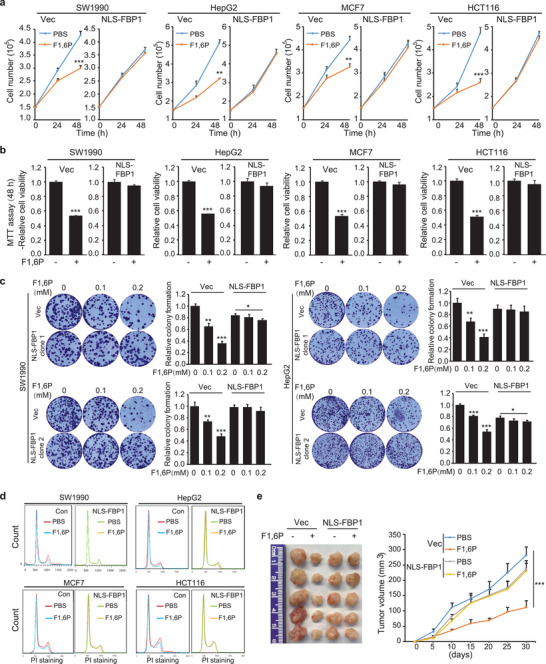
Nuclear accumulation of F1,6P impairs cancer cell viability. a,b) Indicated cancer cells were stably transformed with control or NLS‐FBP1‐expressing vectors and treated with 5 mm F1,6P for the indicated times. a) Cell proliferation was analyzed by counting the cell numbers; b) cell viability was analyzed using an MTT assay. c) The tumor colony formation of indicated cancer cells stably with indicated expression vectors was performed and treated with 0.2 mm F1,6P. The colony number was analyzed using crystal violet staining. d) Indicated cancer cells were stably transformed with control or NLS‐FBP1‐expressing vectors and treated with 5 mm F1,6P. Cell cycle was examined using PI staining and fluorescence‐activated cell sorting (FACS) analyses. e) A total of 4 × 10^6^ SW1990 cells, stably transformed with control or NLS‐FBP1‐expressing vectors, were subcutaneously injected into athymic nude mice, and F1,6P (10 g L^−1^) was injected intraperitoneally every 2 days. Tumor volume was calculated every 5 days (right), and tumor xenografts on day 30 are shown (left). Data represent the means ± s.d. (*n* = 5, **represents *p* < 0.01). In (a–c), the values are presented as mean ± s.d. (*n* = 3), *represents *p* < 0.05, **represents *p* < 0.01, and ***represents *p* < 0.001 with control or the indicated groups. (See also Figure S1, Supporting Information).

The key gluconeogenic enzyme FBP1 acts as a tumor suppressor by depleting cytosolic F1,6P; it is also known to localize to the nucleus and to be involved in transcriptional regulation independent of its enzymatic activity.^[^ ^23^ ^]^ However, the biological role of FBP1's enzymatic activity in the nucleus is unknown. To explore this, cancer cells expressing nuclear‐localized NLS‐FBP1 (Figure S1a–c, Supporting Information) were treated with F1,6P. Unexpectedly, we found that F1,6P treatment dose‐dependently suppressed cell proliferation in various cancer cells (Figure S1d, Supporting Information), but not if the cells overexpressed NLS‐FBP1 (**Figure** **1**a), which consumes nuclear F1,6P and also leads to a slightly compromised cell proliferation by itself. These observations were supported by further cell viability assay (Figure 1b) and tumor colony analysis (Figure 1c and Figure S1e, Supporting Information). In addition, FACS analysis indicated that F1,6P treatment resulted in an appreciable arrest of cells in G1 phase in control but not in NLS‐FBP1 cells (Figure 1d and Figure S1f, Supporting Information).

These unexpected findings seem to contradict F1,6P's role in energetic supply. Aldolase A (ALODA) is the glycolytic enzyme that catalyzes F1,6P conversion. However, after knocking down ALODA, the above effect of F1,6P treatment has not been negated (Figure S1g, Supporting Information), suggesting that the specific effect of F1,6P is largely independent of its role in glycolysis. In line, this effect of F1,6P has also been detected in other cancer cells,^[^ ^24^, ^25^ ^]^ and reactive oxygen species (ROS) induction has been suggested to underlie it.^[^ ^25^ ^]^ However, an inhibitory effect of exogenous F1,6P on ROS level has also been reported^[^ ^8^, ^9^ ^]^ as well as detected in our study (Figure S1h, Supporting Information), suggesting that ROS induction is not the common mechanism in various cancer cells. In any case, our findings here indicate that F1,6P treatment broadly impairs viability of different cancer cells mainly through nuclear‐accumulated F1,6P.

Moreover, some noncancerous cells like NIH3T3 cells, peritoneal macrophages (PM) (Figure S1i, Supporting Information) and primary hepatocyte (Figure S1j, Supporting Information) were also analyzed but without notable effect after F1,6P treatment. Further examination of the intracellular F1,6P level revealed that F1,6P‐sensitive cancer cells including SW1990 and HepG2 accumulated much more F1,6P than did NIH3T3 cells after F1,6P treatment (Figure S1i, Supporting Information). Therefore, we further analyzed F1,6P concentration by mass spectrometry, which confirmed that the F1,6P treatment resulted in a nuclear accumulation in control but not NLS‐FBP1 cancer cells (Figure S1l, Supporting Information). As intracellular F1,6P level is restrained through limited production and unlimited consumption, the above results suggest that to affect cell viability, nuclear F1,6P needs to accumulate to a certain concentration by a direct and sustained supplement such as an exogenous treatment.

F1,6P has been used clinically for ischemia‐related disease. Here, F1,6P administration in nude mice also suppressed tumor development (Figure S1m, Supporting Information and Figure 1e), while this effect in vivo was almost undetectable in NLS‐FBP1 tumors (Figure 1e), suggesting a therapeutic role for F1,6P in cancer treatment. Moreover, no obvious side‐effect of F1,6P administration with the same strategy has been detected after analyzing body weight (Figure S1n, Supporting Information) and key serum markers for liver and kidney functions^[^ ^26^, ^27^, ^28^ ^]^: glutamyl transferase (GGT), alanine aminotransferase (ALT), aspartate aminotransferase (AST), urea nitrogen (BUN) and creatinine (Cr)^[^ ^15^ ^]^ (Figure S1o, Supporting Information). Collectively, these results revealed an anti‐tumor effect of F1,6P through its nuclear accumulation.

### 2.2 Nuclear F1,6P Directly Targets HMGB1

**Figure 2:**
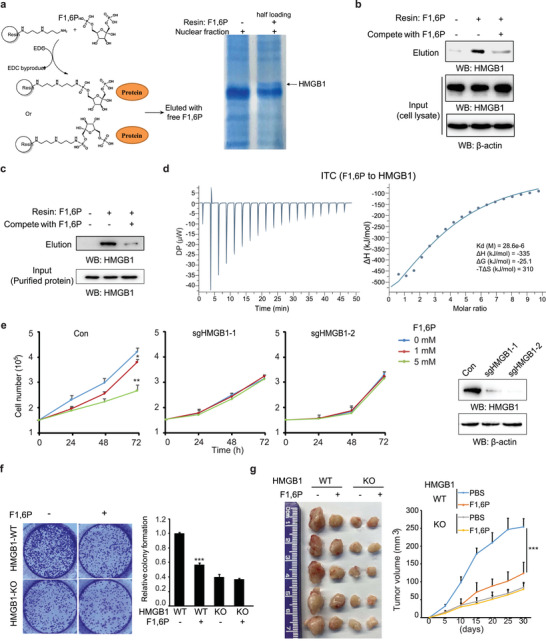
Nuclear F1,6P directly targets HMGB1. a) Left: Schematic diagram showing the strategy for screening of F1,6‐binding proteins. Right: the nuclear fraction of SW1990 cell lysate was incubated with F1,6P‐resin, and the resin‐bound proteins were eluted by free F1,6P and analyzed by Coomassie R‐250 staining and mass spectrometry. b) The cell lysate with or without free 5 mm F1,6P was incubated with F1,6P‐resin, and the resin‐bound proteins were eluted by free F1,6P and analyzed by immunoblotting with the indicated antibodies. c) HMGB1 protein purified from HEK293 cells with or without free 0.8 mm F1,6P in the buffer was incubated with F1,6P‐resin, and the resin‐bound proteins were eluted by free F1,6P and analyzed by immunoblotting with the indicated antibodies. d) The affinity between F1,6P and HMGB1 was measured by isothermal titration calorimetry (ITC), titrating HMGB1 protein with free F1,6P. e) SW1990 cells stably transformed with control or HMGB1 sgRNAs were treated with 0, 1, 5 mm F1,6P, and cell proliferation was analyzed by counting the cell numbers (left); HMGB1 expression was analyzed by immunoblotting (right). f) The tumor colony formation of SW1990 cells stably with control or HMGB1 sgRNA was performed and treated with 0.2 mm F1,6P. The colony number was analyzed using crystal violet staining. g) A total of 6 × 10^6^ SW1990 cells stably with control or HMGB1 sgRNA were subcutaneously injected into athymic nude mice, and F1,6P (10 g L^−1^) was injected intraperitoneally every 2 days. Tumor volume was calculated every 5 days (right), and tumor xenografts on day 30 are shown (left). Data represent the means ± s.d. (*n* = 5, **represents *p* < 0.01). In (e) and (f), the values are presented as mean ±s.d. (*n* = 3), *represents *p* < 0.05, **represents *p* < 0.01 and ***represents *p* < 0.001 with control or the indicated groups. (See also Figure S2, Supporting Information).

To explore the underlying mechanism, an affinity‐based method for unbiased discovery of potential targets of F1,6 in the nucleus was explored. In this method, F1,6P was covalently linked to a primary amine‐functionalized resin to capture F1,6P‐binding proteins in nuclear fractions of cancer cells (**Figure** **2**a, left). The bound proteins were then competitively eluted from the resin using free F1,6P. A specific band was clearly visible only in the elution from F1,6P‐modified resin. The major component of this band was identified to be HMGB1 by mass spectrometry (Figure 2a, right and Excel S1, Supporting Information). Experiments showed that free F1,6P in the cell lysate strongly inhibited HMGB1 binding to the F1,6P‐modified resin (Figure 2b), confirming the specific binding of F1,6P to HMGB1 protein.

Direct interaction between the two was confirmed by showing that purified HMGB1 protein (Figure S2a, Supporting Information) effectively binds to F1,6P‐resin, and that binding was competed by free F1,6P (Figure 2c). Calorimetric measurements were further performed by titrating HMGB1 protein with free F1,6P to characterize the binding properties in vitro. F1,6P showed a binding profile in the isothermal titration calorimetry (ITC) assay with a dissociation constant (K_d_) of 28.6 µm (Figure 2d). Although the concentration of cellular F1,6P can transiently reach a peak as high as several millimoles per liter after stimulation, the physiological concentration of F1,6P is normally low.^[^ ^29^ ^]^ In line with our analysis, the nuclear F1,6P concentration, normally lower than this K_d_ value, can increase highly above it with a sustained supply of exogenous F1,6P (Figure S1l, Supporting Information). It should be noted that F1,6P interacts with the HMGB1 protein ectopically expressed in *Escherichia coli* albeit with lower affinity than the protein purified from mammalian cells (Figure S2b, Supporting Information). Thus, all the full‐length and truncated HMGB1 proteins used in this and the following experiments were expressed and purified from human HEK293 cells (Figure S2a, Supporting Information).

In cells, there is no discernible change in the expression level (Figure S2c, Supporting Information) or subcellular localization (Figure S2d, Supporting Information) of HMGB1 caused by F1,6P treatment. However, an enhanced mobility of GFP‐HMGB1 by F1,6P treatment has been clearly detected in nucleus by fluorescence recovery after photobleaching, at least indicating that F1,6P actually affects the HMGB1 protein in a certain manner in nucleus (Figure S2e, Supporting Information).

Functionally, the inhibitory effect of exogenous F1,6P on cell proliferation was almost fully negated in HMGB1 KO cancer cells (Figure 2e), although the HMGB1 KO obviously affected cell proliferation. F1,6P treatment significantly suppressed colony formation in HMGB1 wild‐type (WT) but not KO cancer cells (Figure 2f and Figure S2f, Supporting Information). In addition, F1,6P administration in nude mice inhibited HMGB1 WT but not KO tumor growth in vivo (Figure 2g). These results indicate that HMGB1 is a direct target of F1,6P in the nucleus, and the major mediator for the anti‐tumor effect of F1,6P treatment.

### 2.3 F1,6P Stabilizes P53 Protein through HMGB1

**Figure 3:**
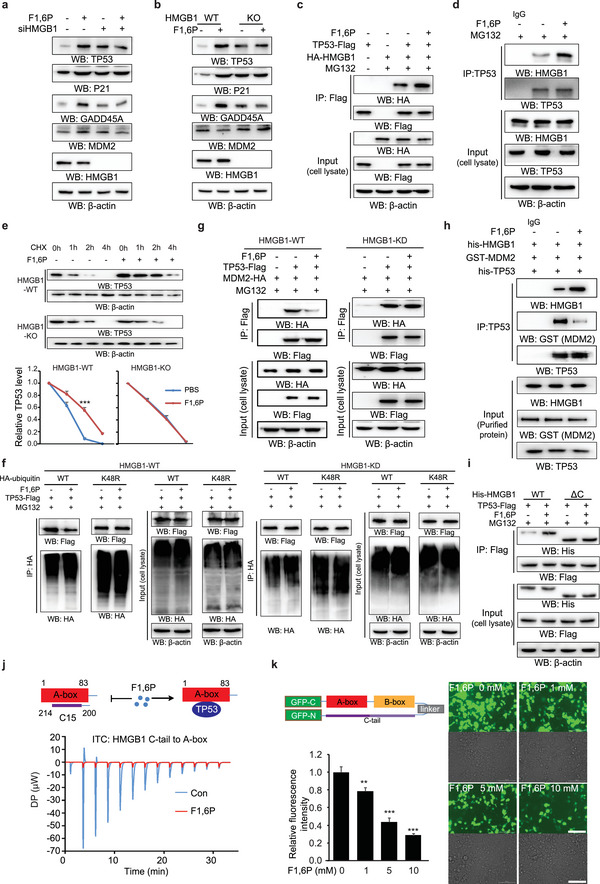
F1,6P stabilizes P53 protein through HMGB1. a) HepG2 cells with the indicated siRNAs and b) sgRNAs were treated with 5 mm F1,6P, and immunoblotting analyses were performed using the indicated antibodies. c,d) SW1990 cells with the indicated expressing vectors pretreated with 20 µm MG132 were treated with 5 mm F1,6P, and cell extracts were immunoprecipitated with c) anti‐Flag or d) anti‐P53 antibody and analyzed by immunoblotting with the indicated antibodies. e) SW1990 cells with the indicated cycloheximide (CHX) and F1,6P treatment were analyzed by immunoblotting with the indicated antibodies. f,g) HEK293 cells with the indicated expressing vectors pretreated with 20 µm MG132 were treated with 5 mm F1,6P, and cell extracts were immunoprecipitated with indicated antibodies and analyzed by immunoblotting with the indicated antibodies. h) The indicated purified proteins treated with 0.2 mm F1,6P in the buffer were subjected to IP with the indicated antibodies and further analyzed by immunoblotting with the indicated antibodies. i) SW1990 cells with the indicated expressing vectors pretreated with 20 µm MG132 were treated with 5 mm F1,6P, and cell extracts were subjected to IP with anti‐Flag antibody and analyzed by immunoblotting with the indicated antibodies. j) The affinity between the HMGB1 A‐box and C‐tail (15) was measured by isothermal titration calorimetry (ITC), titrating purified HMGB1 A‐box with free C‐tail (15) peptide in buffer with or without 0.2 mm F1,6P. k) The fluorescence value of HEK293 cells with the indicated expressing vector and indicated F1,6P treatment in each cell culture well was detected by Cytation cell imaging reader (see also Figure S3i, Supporting Information). Representative images are shown (right); scale bars, 100 µm. Data represent the means ± s.d. (*n* = 3), **represents *p* < 0.01 and ***represents *p* < 0.001 with control groups. (See also Figure S3, Supporting Information).

HMGB1 displays diverse functions, one of which is as a chaperone of the well‐known tumor suppressor P53. Interestingly, F1,6P treatment also markedly upregulates P53 protein (Figure S3a, Supporting Information). GADD45A and P21, which are transcriptionally activated by P53 and implicated in cell cycle arrest, were induced by exogenous F1,6P in WT but not P53 knockdown (KD) cancer cells (Figure S3b, Supporting Information). F1,6P‐mediated increases of P53 as well as GADD45A and P21 were negated in HMGB1 KD (**Figure** **3**a and Figure S3c, Supporting Information) and KO (Figure 3b and Figure S3d, Supporting Information) cancer cells, although the HMGB1 KD/KO also induced P53 expression. Moreover, F1,6P treatment enhanced the cellular HMGB1–P53 association (Figure 3c,d). These results indicate that F1,6P increases P53 protein via HMGB1.

Further experiments revealed that P53 protein was stabilized after F1,6P treatment (Figure 3e and Figure S3e, Supporting Information). P53 degradation is typically signaled by MDM2‐mediated K48 polyubiquitination;^[^ ^30^ ^]^ and we found that F1,6P treatment inhibited cellular P53 modification with WT but not K48R ubiquitin, which was negated in HMGB1 KD cells (Figure 3f). Concordantly, F1,6P treatment weakened the cellular P53–MDM2 interaction in HMGB1 WT but not KD cells (Figure 3g), but had no effect on the interaction between the purified proteins (Figure S3f, Supporting Information). MDM2 binding to the amino acid residues (aa) 19–26 of P53 is important for P53 degradation.^[^ ^31^ ^]^ HMGB1 can interact with the same and adjacent residues of P53 with lower affinity.^[^ ^20^ ^]^ HMGB1 is an abundant protein; thus, the enhanced HMGB1–P53 affinity in the presence of F1,6P seemed likely to preclude MDM2–P53 interaction, which was proved with an IP analysis using purified HMGB1, MDM2, and P53 proteins (Figure 3h). These results satisfactorily explain how F1,6P stabilizes P53 protein through HMGB1.

HMGB1 protein consists of three domains: two basic HMG boxes (A‐ and B‐boxes) and an acidic C‐tail (Figure S3g, Supporting Information). P53 can independently interact with both purified A‐ and B‐box in vitro, but interacts with full‐length HMGB1 monomer with a 1:1 stoichiometry by preferentially contacting the A‐box only.^[^ ^32^, ^33^ ^]^ However, the P53 binding site in the A‐box is dynamically sequestered by the C‐tail.^[^ ^20^ ^]^ Consistent with this, a C‐tail‐truncated HMGB1 protein (HMGB1‐ΔC) associated with more P53 than did full‐length HMGB1, and the truncation compromised the stimulatory effect of F1,6P on HMGB1–P53 interaction (Figure 3i). Thus, we speculate that binding with F1,6P affects the affinity between the HMGB1 A‐box and C‐tail, presumably by exposing more A‐box for P53 access (Figure 3j, upper). To test this hypothesis, we purified HMGB1 A‐box (aa1–83) (Figure S3h, Supporting Information) and synthesized the C15 peptide (aa200–214). Subsequent ITC analysis indicated that C15 peptide displayed good affinity for the A‐box, whereas the interaction was highly disrupted by F1,6P (Figure 3j, lower). To test this effect of F1,6P in live cells, we developed a split‐GFP‐based bimolecular fluorescence complementation (BiFC) assay to evaluate the conformational change of HMGB1. We generated a construct expressing GFP(C)‐HMGB1(A‐B)‐linker‐HMGB1(C)‐GFP(N) fusion protein (Figure 3k upper), which will fluoresce once the A‐box and C‐tail bring GFP(N) and GFP(C) into close proximity. Experiments showed that the expression level of the fusion protein was not affected by F1,6P treatment (Figure S3i, Supporting Information), but the fluorescence signal, which is an indicator of A‐box–C‐tail association, was clearly compromised by F1,6P treatment (Figure 3k). Collectively, these results indicate that F1,6P lowers the affinity between the HMGB1 A‐box and C‐tail, resulting in enhanced HMG A‐box–P53 interaction and P53 stabilization.

### 2.4 F1,6P Disrupts the HMGB1 Oligomer

**Figure 4:**
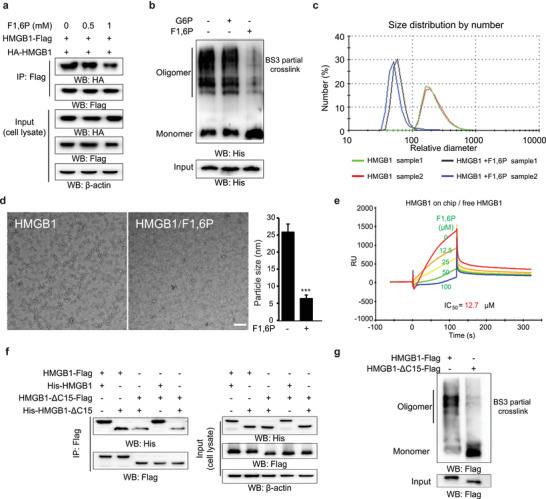
F1,6P disrupts the HMGB1 oligomer. a) HEK293 cells with the indicated expressing vectors were treated with indicated concentration of F1,6P, and cell extracts were subjected to IP with anti‐Flag antibody and analyzed by immunoblotting with the indicated antibodies. b) Purified HMGB1 protein in the buffer with F1,6P or G6P was exposed to BS3 crosslinker (0.1 mm for 10 min) and further analyzed by immunoblotting with anti‐His antibody. c) Relative particle size of purified HMGB1 protein (independently purified protein sample1 and 2) in buffer with or without 0.2 mm F1,6P was measured by the dynamic light scattering assay. d) Morphology of purified HMGB1 protein in buffer with or without 0.2 mm F1,6P was detected by electron microscopy. Scale bar: 100 nm. e) Surface plasmon resonance (SPR) of free HMGB1 protein with serially diluted F1,6P (concentrations indicated) binding to chip‐anchored HMGB1 was analyzed on a Biacore platform. f) SW1990 cells with the indicated expressing vectors were treated with 5 mm F1,6P, and cell extracts were subjected to IP with anti‐Flag antibody and analyzed by immunoblotting with the indicated antibodies. g) The indicated full‐length or truncated HMGB1 proteins were exposed to BS3 crosslinker (0.1 mm for 10 min) and further analyzed by immunoblotting with anti‐Flag antibody. (See also Figure S4, Supporting Information).

HMGB1 protein originally purified from rat liver existed as an oligomer in physiological buffer and in stable complex with ssDNA.^[^ ^13^ ^]^ However, in almost all subsequent biochemical analyses using protein purified from *E. coli*, HMGB1 mainly existed as a monomer.^[^ ^20^, ^32^, ^34^ ^]^ Here, using cancer cells expressing epitope‐tagged HMGB1 or protein purified from human cells, we independently showed that HMGB1 can spontaneously self‐associate into multimers but that these can be further disrupted by F1,6P: first, HA‐tagged HMGB1 was co‐immunoprecipitated with Flag‐tagged HMGB1, which was dose‐dependently suppressed by F1,6P treatment (**Figure** **4**a); second, purified HMGB1 protein was partially crosslinked after a brief exposure to BS3 only in F1,6P‐free buffer (Figure 4b); third, the dynamic light scattering assay, a physical approach for measuring particle size, also indicated that F1,6P substantially decreased the relative size of purified HMGB1 protein (Figure 4c); fourth, these observations were further confirmed by directly monitoring HMGB1 protein under electron microscopy, which clearly showed much smaller particle size after incubation with F1,6P (Figure 4d). Additionally, surface plasmon resonance (SPR) analysis was performed to quantify the inhibitory efficiency of F1,6P on free HMGB1 binding to chip‐anchored HMGB1 (Figure S4a, Supporting Information), yielding an IC_50_ value of 12.7 µm (Figure 4e).

F1,6P decreases the affinity between the A‐box and C‐tail (Figure 3i) and also disrupts HMGB1 oligomerization (Figure 4a–f), suggesting that A‐box–C‐tail association contributes to HMGB1 oligomerization. We tested this hypothesis using the HMGB1‐ΔC protein with a pull‐down assay. The results indicated that HMGB1 self‐association occurred strongly between two WT proteins or WT and ΔC proteins but weakly between two ΔC proteins (Figure 4f). Partial crosslinking analysis also indicated a much weaker affinity between purified HMGB1‐ΔC proteins (Figure 4g), suggesting that the intermolecular A‐box–C‐tail association was one of the main contributors for HMGB1 oligomerization (Figure S4b, Supporting Information). Collectively, these results show that binding with F1,6P directly de‐oligomerizes HMGB1.

### 2.5 F1,6P Impairs DNA‐Associated Functions of HMGB1

**Figure 5:**
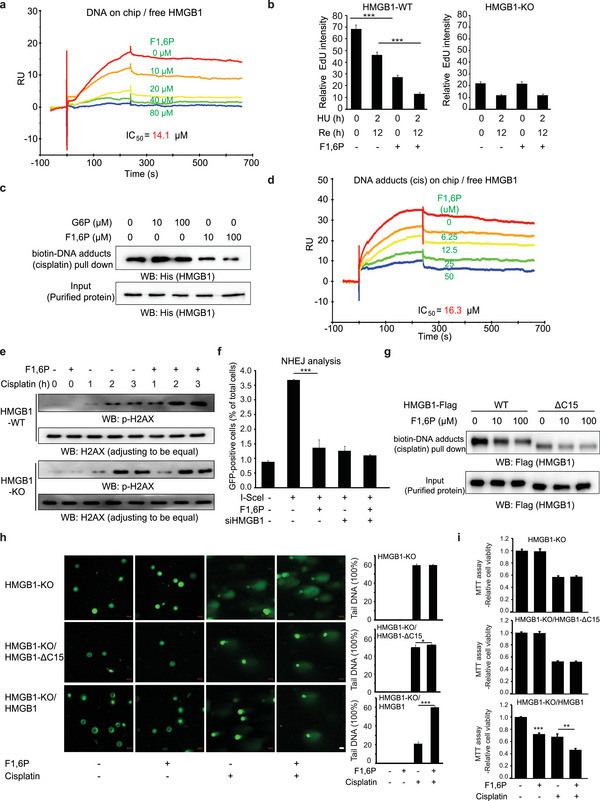
F1,6P impairs DNA‐associated functions of HMGB1. a) SPR of free HMGB1 protein with serially diluted F1,6P (concentrations indicated) binding to chip‐anchored DNA was analyzed on a Biacore platform. b) DNA replication efficiency of SW1990 cells with the indicated HU and F1,6P treatments was analyzed by EdU incorporation. EDU intensity in EDU positive cells was quantified. c) Purified HMGB1 protein in buffer with F1,6P or G6P binding to cisplatin‐treated biotin‐DNA was analyzed by streptavidin pull‐down and further immunoblotting analyses with the indicated antibody. d) SPR of free HMGB1 protein with serially diluted F1,6P (concentrations indicated) binding to chip‐anchored and cisplatin‐treated DNA was analyzed on a Biacore. e) HMGB1 WT‐ or KO‐transformed SW1990 cells with the indicated cisplatin and F1,6P treatments were analyzed by immunoblotting with the indicated antibodies. f) The efficiency of rejoining of I‐SceI‐induced double‐strand DNA breaks in U2OS cells with the indicated treatments was analyzed by FACS. g) The indicated full‐length or truncated HMGB1 proteins in buffer with F1,6P binding to cisplatin‐treated biotin‐DNA were analyzed by streptavidin pull‐down and immunoblotting with anti‐Flag antibody. h,i) HMGB1 KO SW1990 cells with the indicated expressing vectors were treated as indicated cisplatin and F1,6P. h) DNA damage was analyzed by comet assay and representative images (scale bars, 20 µm) are shown; i) cell viability was analyzed using an MTT assay. In (b), (f), (h), and (i), the values are presented as mean ± s.d. (*n* = 3), *represents *p* < 0.05, and *** represent *p* < 0.001 with control or the indicated groups. (See also Figure S5, Supporting Information).

As the most abundant non‐histone chromosome structural protein, HMGB1 is important for various DNA‐associated processes. To test whether F1,6P affects these functions of HMGB1, SPR analysis for free HMGB1 binding to chip‐anchored DNA was performed and the results indicated an effective HMGB1–DNA interaction (Figure S5a, Supporting Information), which was compromised by F1,6P with an IC_50_ value of 14.1 µm (**Figure** **5**a). Although no evidence, there is possibility that F1,6P increasing the mobility of HMGB1 protein in nucleus (Figure S2e, Supporting Information) may be caused by the decreased HMGB1–DNA association and HMGB1 self‐association (Figure 4).

HMGB1 is known to support DNA replication,^[^ ^35^, ^36^ ^]^ which was analyzed by EdU incorporation assay (the quantification of EDU intensity only in EDU positive/S stage cells) (Figure 5b and Figure S5b, Supporting Information). Treatment with the chemotherapy drug hydroxyurea (HU) fully arrested the process of DNA replication, which recovered 12 h after HU was removed. F1,6P effectively suppressed the recovery of DNA replication in HMGB1 WT but not in KO cells, indicating that it impairs DNA replication by targeting HMGB1.

Since HMGB1 preferentially binds to DNA modified with carcinogens or chemotherapeutic agents, the roles of HMGB1 in various DNA repair pathways have been studied in numerous reports, in which binding of HMGB1 to the damaged DNA was proposed to be among the initial steps during the DNA damage response (DDR) and serves to alter DNA structure or to facilitate the recruitment of various factors involved in DDR. Thus, a pull‐down assay was performed to analyze the binding of HMGB1 to cisplatin‐modified DNA adducts, which was also suppressed by F1,6P but not G6P (Figure 5c). The IC_50_ for the inhibition of the HMGB1–DNA adducts (cisplatin) interaction by F1,6P was also determined by SPR analysis to be 16.3 µm (Figure 5d). Consistent with these observations, a comet assay showed that F1,6P dramatically aggravated cisplatin‐induced DNA damage as well as DNA replication stress (HU)‐induced damage (Figure S5c, Supporting Information). *γ*H2AX, a widely used marker for DNA damage, is induced by cisplatin. However, F1,6P notably decreased both basal and cisplatin‐induced cellular *γ*H2AX levels (Figure S5d, Supporting Information), which seems to be inconsistent with the results from the comet assay. Further analysis revealed that the total H2AX protein levels were also suppressed by either F1,6P treatment or HMGB1 KO (Figure S5e, Supporting Information). After adjusting the loading to ensure that the total H2AX level was equal in each lane, we verified that F1,6P treatment remarkably increased the cisplatin‐induced phosphorylation of H2AX in HMGB1 WT but not in KO cells (Figure 5e). HMGB1 has been reported to be involved in various DNA repair pathways including non‐homologous end joining (NHEJ), which is a major contributor in repair of DNA damage induced by chemotherapy. Therefore, we performed a NHEJ reporter assay based on rejoining I‐SceI‐induced double‐strand DNA breaks (DSBs). This assay showed that F1,6P significantly suppresses the repair of DSBs by NHEJ in HMGB1 WT but not in KO cancer cells (Figure 5f and Figure S5f, Supporting Information).

In addition, compared to WT HMGB1, HMGB1‐ΔC displayed a much weaker affinity for cisplatin‐modified DNA adducts, which was slightly affected by F1,6P (Figure 5g). Moreover, in HMGB1‐KO cells, the comet assay indicated that F1,6P had an insignificant effect on cisplatin‐induced DNA damage but appreciably aggravated DNA damage in cells re‐expressing WT but not ΔC HMGB1 (Figure 5h), which is consistent with the combined inhibitory effect of cisplatin and F1,6P on cell viability (Figure 5i). Additionally, in P53 null Hep3B and H1299 cancer cells (Figure S5g, Supporting Information), the aggravated cisplatin‐induced DNA damage (Figure S5h, Supporting Information) and cell viability (Figure S5i, Supporting Information) by F1,6P treatment were also notably detected. Meanwhile, F1,6P treatment alone also impaired the cell viability in these cancer cells (Figure S5i, Supporting Information). These results suggested that the functional readout of F1,6P‐HMGB1 interaction should be mainly contributed by impaired DNA replication and DNA repair, especially in P53 null or mutated cancer cells. Collectively, all these results indicate that F1,6P aggravates chemotherapy drug‐induced DNA replication stress and DNA damage by targeting HMGB1.

### 2.6 K43/K44 Residues of HMGB1 Mediate the Anti‐Tumor Effect of F1,6P

**Figure 6:**
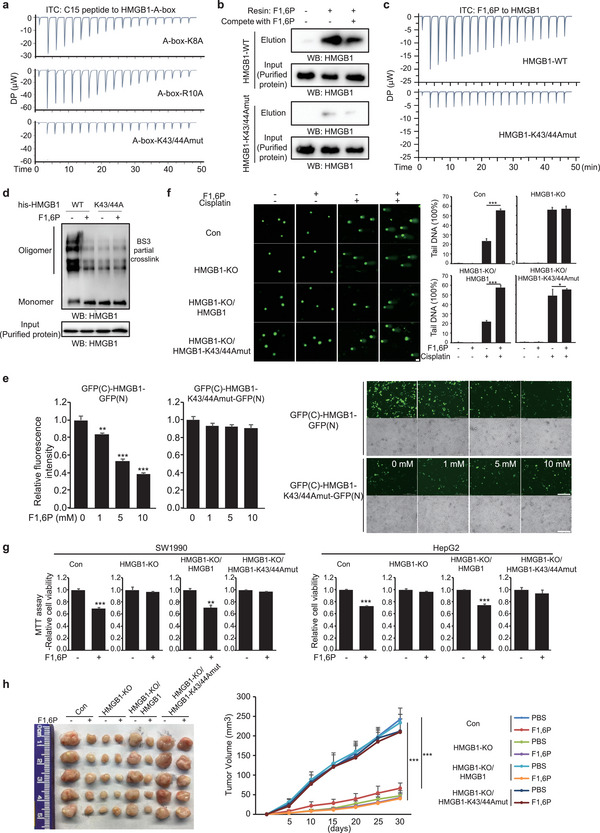
K43/K44 residues of HMGB1 mediate the anti‐tumor effect of F1,6P. a) The affinity between the indicated mutated A‐boxes and C‐tail was measured by ITC, titrating purified HMGB1 A‐box with free C‐tail (15) peptide. b) Indicated HMGB1s protein purified from HEK293 cells with or without free 0.8 mm F1,6P in the buffer were incubated with F1,6P‐resin, and the resin‐bound proteins were eluted by free F1,6P and analyzed by immunoblotting with the indicated antibodies. c) The affinity between F1,6P and WT or mutated HMGB1 was measured by ITC, titrating HMGB1 protein with free F1,6P. d) The indicated HMGB1 proteins were exposed to BS3 crosslinker (0.1 mm for 10 min) and further analyzed by immunoblotting with anti‐HMGB1 antibody. e) The fluorescence value of HEK293 cells with the indicated expressing vector and indicated F1,6P treatment in each cell culture well was detected by Cytation cell imaging reader (see also Figure S6d, Supporting Information). Representative images are shown (right); scale bars, 200 µm. f) SW1990 cells transformed with the indicated HMGB1 expression vectors were treated with cisplatin and F1,6P as indicated, and DNA damage was analyzed by comet assay. Representative images are shown; scale bars, 20 µm. g) The indicated cancer cells stably transformed with the indicated HMGB1 expression vectors were treated with 5 mm F1,6P, and cell viability was analyzed using an MTT assay. h) A total of 3 × 10^6^ SW1990 cells stably transformed with the indicated HMGB1 expression vectors were subcutaneously injected into athymic nude mice, and F1,6P (10 g L^−1^) was injected intraperitoneally every 2 days. Tumor volume was calculated every 5 days (right), and tumor xenografts on day 30 are shown (left). Data represent the means ± s.d. (*n* = 5, ***represents *p* < 0.001) In (e–g), the values are presented as mean ± s.d. (*n* = 3), *represents *p* < 0.05, **represents *p* < 0.01 and ***represents *p* < 0.001 with control or the indicated groups. (See also Figure S6, Supporting Information).

The association between the HMGB1 A‐box and C‐tail is disrupted by F1,6P (Figure 3i), but how the A‐box and C‐tail are associated is unclear. The structure of HMG box is known;^[^ ^17^, ^33^ ^]^ otherwise, HMGB1 protein structure is unclear but has recently been predicted by AlphaFold2 with high confidence^[^ ^37^ ^]^ (Figure S6a, Supporting Information). Several positively charged residues like K8, R10, and K43/K44 in the A‐box may contribute to acidic C‐tail binding, and these residues were mutated to uncharged alanine. ITC analysis indicated that only the purified K43/44A A‐box bound to C‐tail with a much lower affinity (**Figure** **6**a and Figure S6b, Supporting Information), suggesting the K43/K44 residues contribute to A‐box–C‐tail association. Interestingly, the K43/44A mutation also dramatically impaired the affinity between F1,6P and HMGB1 that was analyzed by F1,6P‐resin pull down (Figure 6b and Figure S6c, Supporting Information) and ITC (Figure 6c), indicating that these residues contribute to the binding with negatively charged F1,6P. BS3 crosslinking analysis also indicated a weak affinity between HMGB1‐K43/K44A proteins, which was almost unaffected by F1,6P (Figure 6d). We further generated the indicated GFP(C)‐HMGB1‐K43/K44A‐GFP(N) construct as the in vivo indicator of A‐box–C‐tail association in Figure 3j; and their expression levels were not affected by F1,6P treatment (Figure S6d, Supporting Information). Compared to the WT fusion protein, the K43/K44A mutation dramatically impaired the fluorescence signal, which was only slightly affected by F1,6P treatment (Figure 6e). These findings well explain how F1,6P disrupts the A‐box–C‐tail association. Consistent with this, the HMGB1 K43/44A mutation (K43/44Amut) associated with more P53 than did HMGB1 WT, and the mutation compromised the stimulatory effect of F1,6P on HMGB1–P53 interaction (Figure S6e, Supporting Information).

Additionally, F1,6P enhanced cisplatin‐induced DNA damage in HMGB1‐KO cancer cells with re‐expressed WT but not K43/44A HMGB1 (Figure 6f). In agreement, cell proliferation (Figure S6f, Supporting Information) and cell viability (Figure 6g) were significantly inhibited in HMGB1‐KO cancer cells with re‐expressed WT but not K43/44A HMGB1. Moreover, F1,6P administration in nude mice inhibited WT but not K43/44A HMGB1 tumor growth in vivo (Figure 6h). Collectively, these results indicate that the K43/44 residues of HMGB1 mediate the anti‐tumor effect of F1,6P.

### 2.7 F1,6P Could Be an HMGB1‐Targeting Drug for Cancer, Alone or in Combination with Chemotherapy

**Figure 7:**
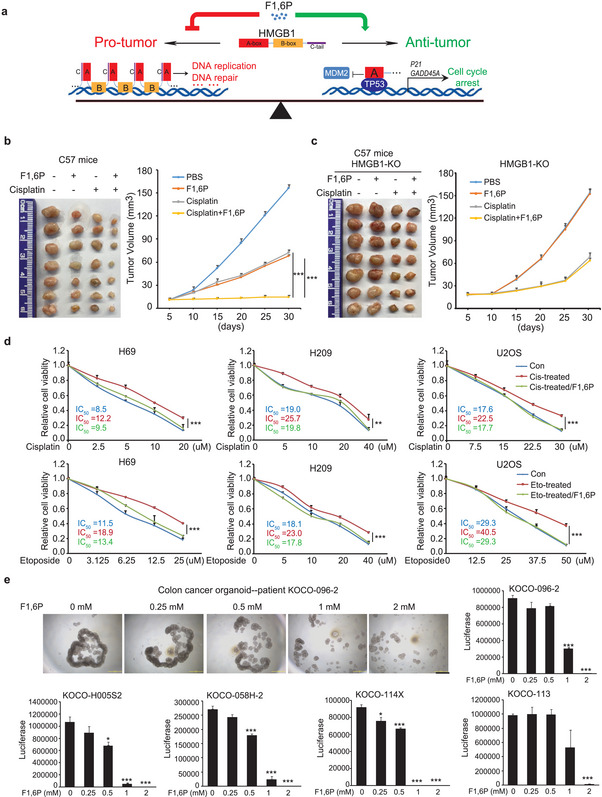
F1,6P could be an HMGB1‐targeting drug for cancer therapy. a) Schematic model showing the anti‐tumor role of F1,6P through its binding to HMGB1. A total of b) 2 × 10^6^ Pano2 cells with or c) 5 × 10^6^ Pano2 cells without HMGB1 were subcutaneously injected into C57 mice, and cisplatin and F1,6P were injected intraperitoneally every 2 days. Tumor volume was calculated every 5 days (right), and tumor xenografts on day 30 are shown (left). Data represent the means ± s.d. (*n* = 7 mice per group, ***represents *p* < 0.001). d) Indicated cancer cells with or without cisplatin or etoposide pretreatment were treated with cisplatin and etoposide as indicated with or without 0.5 mm F1,6P. Cell viability was analyzed using an MTT assay. e) The effect of F1,6P treatment on colon cancer organoids from different patients was analyzed using a luciferase‐based assay. Representative images are shown; scale bars, 500 µm. (See also Figure S7, Supporting Information).

HMGB1 is elevated in multiple tumors, but paradoxical roles of HMGB1 in tumor development have been reported. Here, we found that F1,6P promotes anti‐tumor functions (like P53 activation) but inhibits pro‐tumor functions (like DNA replication and repair) of HMGB1 in the nucleus (**Figure** **7**a). Upon targeting HMGB1, F1,6P aggravates chemotherapy drug‐induced DNA replication stress and DNA damage (Figures 5 and 6), suggesting that F1,6P would sensitize chemotherapy. Consistent with this idea, F1,6P treatment significantly enhanced cisplatin‐induced apoptosis with a synergetic effect (38%) more than a simple additive effect (21%) in WT (Figure S7a, Supporting Information) but not in HMGB1‐KO (Figure S7b, Supporting Information) cancer cells. Moreover, the combined inhibitory effect of cisplatin and F1,6P on cell viability was also detected in HMGB1 WT but not KO Pano2 (mouse) cancer cells (Figure S7c, Supporting Information). F1,6P administration in C57 mice also synergistically enhanced the therapeutic effect of cisplatin on WT (Figure 7b) but not HMGB1‐KO (Figure 7c) tumors (Pano2) in vivo.

Chemotherapy resistance is the major impediment to the clinical treatment of, especially, small cell lung cancer (SCLC) and multiple myeloma (MM). Therefore, the SCLC H209, H69 (P53 mutated) cells and MM U2OS cells with cisplatin and etoposide resistance have been generated through chronic pre‐treatment with these two commonly used chemotherapeutic drugs. Moreover, F1,6P, at a lower dose without significant effect by itself (Figure S7d, Supporting Information), can significantly rescue the sensitivity to both cisplatin and etoposide in all these cancer cells (Figure 7d). Moreover, F1,6P also resensitized P53 null cancer cells (Hep3B and H1299) with chemotherapy resistance (Figure S7e, Supporting Information).

More interestingly, Multiple colon tumor organoids were established from clinical samples of different patients. To our surprise, F1,6P treatment alone was sufficient to eliminate these human tumor organoids, at a lower dose compared to cancer cell lines (Figure 7e and Figure S7f, Supporting Information). Collectively, all these results suggest an interesting and promising role for F1,6P as an HMGB1‐targeting drug for cancer therapy, alone or in combination with chemotherapy.

## 3 Conclusions

Cell metabolism supports and regulates almost all biological activities. The metabolites are evolutionarily selected as natural regulators for various biological events that are either dependent on or independent of metabolic reactions. Thus, endogenous metabolites and their derivatives should be an important source for drug exploration. In this study, we discovered a non‐metabolic protein that binds to F1,6P in the nucleus of mammalian cells. We found that F1,6P is an allosteric ligand of the chromosome structural protein HMGB1 and behaves as a potential and promising anti‐tumor drug. Thus, chemical modification to generate F1,6P derivatives with higher anti‐tumor activity could be explored in further research.

Metabolites need to be maintained at an appropriate level to support cellular demands. Our study with F1,6P illustrates a well‐established paradigm that either too high or too low a level of a specific metabolite in cells may bring unexpected biological effects. Moreover, our study indicates that both the extracellular supply and the cellular uptake ability are important for nuclear F1,6P accumulation. Therefore, further research should now be carried out to clarify the mechanism of F1,6P uptake in tumor cells, which may foster the development of a better strategy for cancer therapy with F1,6P.

HMGB1 is often considered as a cancer therapeutic target but with many challenges. There are several advantages for utilizing F1,6P to target HMGB1: First, HMGB1 KO mice are lethal, but F1,6P administration in mice has no obvious side effect; second, F1,6P treatment usually play a cytotoxic effect in cancer cells but a cytoprotective effect in various primary cells; third, HMGB1 is elevated in multiple tumors but plays paradoxical roles. F1,6P promotes the anti‐tumor (P53 activation) but inhibits the pro‐tumor functions (DNA replication and repair) of HMGB1.

Histones and HMGB proteins belong to the two major families of chromosome structural proteins, which are important for various DNA‐associated processes including transcription, DNA replication, and DNA repair. Established mechanisms for DNA structural regulation are mainly based on modifications of DNA and DNA structural proteins, such as methylation. The modifying enzymes can be affected by certain metabolites in a concentration‐dependent manner, resulting in metabolic regulation of epigenetics. Alternatively, here, we have shown that the metabolite F1,6P can also directly bind to the DNA structural protein HMGB1 as an allosteric ligand to influence DNA‐associated process.

Most previous in vitro studies concerning the HMGB1 protein were carried out using proteins expressed in *E. coli*. In our study, we noticed that purified HMGB1 proteins isolated from *E. coli* and human cells did not exhibit the same properties (for example, the oligomeric state). This observation suggests that post‐translational modification plays far more important roles in HMGB1 function than previously thought. Experiments using HMGB1 protein isolated from mammalian cells should produce interesting findings that take more account of HMGB1 with post‐translational modification.

In this work, we only investigated the biological importance of the intracellular F1,6P–HMGB1 interaction. However, HMGB1 performs diverse functions in different locations: for example, HMGB1 also functions as an extracellular inflammatory cytokine.^[^ ^21^ ^]^ More interestingly, the elevated HMGB1 level in blood is considered as a biomarker and an effective therapeutic target for many inflammatory and autoimmune diseases;^[^ ^38^ ^]^ on the other hand, F1,6P administration has shown an anti‐inflammatory effect in mice.^[^ ^39^ ^]^ Therefore, further research to explore the biological role of the interaction between F1,6P and HMGB1 in the extracellular environment may shed more light on the pharmacological importance of F1,6P.

Our study also suggested that a high level of intracellular F1,6P would adversely affect the DNA‐related physiological functions of HMGB1. Coincidentally, the most important physiological function of HMGB1 is to support the transcription of gluconeogenic genes. As shown in a previous report, HMGB1 KO mice exhibited abnormal hepatic glycogen accumulation and lethal hypoglycemia.^[^ ^11^ ^]^ F1,6P is the rate‐limiting substrate in the gluconeogenic pathways, and intracellular F1,6P can be regarded as an indicator for the availability of blood sugar. Therefore, it is possible that glycolysis can intersect with the transcription of gluconeogenic genes through the intracellular F1,6P–HMGB1 interaction. The physiological role of the F1,6P–HMGB1 interaction in transcriptional regulation is also an intriguing topic in need of further investigation.

## 4 Experimental Section

### Cell Culture and Stable Cell Line Generation

All cell lines were obtained from the National Infrastructure of Cell Line Resource of China or gifted from the professors of Tianjin Medical University, and routinely confirmed to be mycoplasma free. HEK293T, HCT116, HepG2, MCF7, NIH3T3, U2OS, PanO2, H209, H69, Hep3B, and H1299 cells were cultured in Dulbecco's Modified Eagle Medium (DMEM, Gibico, C11885500BT) supplemented with 10% heat‐inactivated fetal bovine serum (FBS, Gibico, 10270‐106), 100 units mL^−1^ penicillin and 100 µg mL^−1^ streptomycin (Solarbio, P1400), non‐essential amino acids (Solarbio, N1250). SW1990 cells were grown in RPMI1640 (Gibico, C11875500BT) supplemented with 10% FBS, 100 units mL^−1^ penicillin, and 100 µg mL^−1^ streptomycin. They were maintained in a humidified 5% CO_2_/95% air atmosphere at 37 °C.

Stable cell lines were generated via a lentiviral delivery system. The lentiviral sgRNA vectors were generated via ligation of hybridized oligos into lentiCRISPR‐v2 vector linearized with BsmBI‐v2 (NEB, R0739)) using T4 DNA ligase (NEB, M0202). Infected cells were selected with 2 µg mL^−1^ puromycin (Solarbio, P8230) for at least 3 days and whole‐cell extracts were then used for immunoblot analysis to confirm the integration of the transgenes or knock out in the genome. To increase homogeneity within cell lines, monoclonal cell lines were generated by isolating single colonies from culture plates seeded with appropriately diluted cell suspension.

### Plasmids, siRNAs, and Transfection

Plasmid encoding HA‐Ubiquitin, HA‐Ubiquitin‐K48, HA‐Ubiquitin‐K48R were purchased from Addgene and used directly without modification. Plasmid encoding pBiFC‐VC155, pBiFC‐VN173, MDM2‐YFP were purchased from Addgene and used as templates for subcloning. The plasmids were constructed for this study as described in “KEY RESOURCES TABLE” (Supporting Information). All plasmid inserts were validated by sequencing at Genewiz. All siRNAs were purchased from Synbio Technologies. The sequences of the siRNA were described in “KEY RESOURCES TABLE” (Supporting Information). Cultured cells were transfected with different plasmids using Lipofectamine 2000 (Invitrogen, 11668‐019) and with siRNAs using RNAi Max (Invitrogen, 13778150) according to the manufacturer's instructions.

### Cell Proliferation and Viability Assay

For cell proliferation, cells at a density of 1.5 × 10^5^/well were seeded in 12‐well plates. After treatment, cells were harvested and determined cell number using trypan blue (Solarbio, C0040) stain by cell counting plate. For MTT assay, cells were seeded in 96‐well plate with a density of 5 × 10^3^ per well in 200 uL of complete media. After treatment, the cultures were washed with PBS (phosphate buffered solution, Solarbio, P1022) for three times. Cytotoxicity was assessed using MTT (3‐[4,5‐dimethylthiazol‐2yl]‐2,5‐diphenyltetrazolium bromide, Solarbio, M8180). MTT solutions 20 uL (5 mg mL^−1^ in PBS) along with 200 uL of fresh, complete media were added to each well, and plates were incubated for 4 h. After incubation, add 200 µL DMSO (Solarbio, D8371) into each well. Wrap plate in foil and shake on an orbital shaker for 5 min. Occasionally, pipetting of the liquid may be required to fully dissolve the MTT formazan. Absorbances were measured using microplate reader at 490 nm and results were expressed as % viability which was directly proportional to metabolic active cell number. Percentage (%) viability was calculated as: Cell Viability (100%) = OD in treatment well/OD in control(PBS) well × 100.

For CCK8 assay, the cell proliferation was measured using the Cell Counting Kit‐8 (NCM Biotech, C6005). The transfected cells were plated into 96‐well plates at a density of 5 × 10^3^/well/100 µL. and then 10 µL of CCK8 solution was added to each well, followed by incubation for 2 h. The cell viability was determined by measuring the absorbance at 450 nm.

### Colony Formation Assay

HepG2 or SW1990 cells were harvested after trypsinization, counted, and plated at a density of 250 cells well^−1^ (6‐well plate). The medium was refreshed once every 4 days. 2 weeks later, cells were washed in 1× PBS twice, fixed in 4% Paraformaldehyde Fix Solution (Paraformaldehyde, Sigma Aldrich, 158127), stained with 0.1% crystal violet (Solarbio, G1064) and then counted. The cell colony was counted (each colony contains at least 50 cells).

### Cell Cycle and Apoptosis Analysis

Cells at around 80% confluency were harvested and pelleted by centrifugation. The supernatant was discarded, the tube was tapped to resuspend the pellet in the residual liquid and one volume of PBS was added, three volume of chilled ethanol was mixed and left to fix overnight at 4 °C. The fixed cells were pelleted, washed twice with PBS containing 1% BSA (Albumin Bovine, Solarbio, A8010), and stained with 50 ug mL^−1^ propidium iodide (Invitrogen, P1304MP) for 30 min with rotation in the dark at room temperature. Rnase was added with continued rotation for another 30 min. The cell cycle distribution was analyzed by flow cytometry on BD FACSVerse in the presence of the dye. Data were analyzed using Flowjo 7.6.1. For data analysis, cell debris was first gated out by finding the largest subpopulation using SSC‐A and FSC‐A. Subsequently, singlet cells were selected by gating on the basis of PI‐A versus PI‐W. These two gates were applied to all samples and each cell sample was analyzed further. Apoptosis was assessed by Annexin‐V/PI double staining (Annexin V‐FITC Cell Apoptosis Analysis Kit, SungeneBiotech, AO2001‐02P). Cells (≈1 × 10^6^ cells per test) were harvested with trypsin and then washed with cold PBS. Cells were suspended in 1 mL 1× Binding Buffer, centrifuged at 300 × *g* for 10 min, and the Binding Buffer was then removed from the cell pellet. Cells were resuspended in 1 mL 1× Binding Buffer, cell concentration was adjusted to 1 × 10^6^ cells mL^−1^. 100 uL of cells (1 × 10^5^ cells) were added to each labeled tube. 5 uL of Annexin V‐FITC was added to appropriate tubes. Each tube was gently vortexed and incubated for 10 min at room temperature, protected from light. PBS was added to 500 uL and was gently vortexed. Analysis was done by flow cytometry (BD FACSVerse) in 1 h.

### ROS Assay

The intracellular ROS levels were detected by Reactive Oxygen Species Assay Kit (BeyotimeBeyotime, S0033S). 2′, 7′‐dichlorofluorescein‐diacetate (DCFH‐DA) was easily oxidized to fluorescent dichlorofluorescein (DCF) by intracellular ROS. Therefore, the level of reactive oxygen species in the cell can be known by detecting the fluorescence of DCF. The ROS levels were quantified. Briefly, the cells were incubated with 10 uM DCFH‐DA for 20 min at 37 °C and then observed using fluorescence microscopy and measured at 488 nm excitation and 525 nm emission.

### F1,6P Assay

Cells were treated with or without 5 mm F1,6P (D‐fructose 1,6‐diphosphate sodium, Macklin, D832357) for 24 h. Cell pellets were washed with PBS at least for five times. After cell disruption, NADH (17 uM) was added to the samples. The sample was thoroughly mixed and then added GDH 130 U/L and TIM 830 U/L. Incubations were performed at room temperature for 10 min. After the reaction occurred for 10 min, absorbance was read (340 nm) A_1_. Then, Aldolase 45 U/L was added to each sample. After the reaction has occurred for 10 min, read absorbance (340 nm) A_2_; A_1_ − A_2_ = △A_F1,6P._

### Tumor Xenografts

C57BL/6J mice and BALB/c nude mice for tumor xenografts were purchased from Beijing Vital River Laboratory Animal Technology Co. Ltd. All animals were kept under specific pathogen free and temperature‐controlled environment with 12 h light/12 h dark cycle, and free access to food and water. All animal studies were performed under the guidelines of the Animal Care and Use Committee of Tianjin Medical University and approved by the Ethics Committee of Tianjin Medical University (TMULA‐2018030). ≈6‐week‐old male C57BL/6J mice were injected with 2 × 10^6^ Pan02 (WT) cells or 5 × 10^6^ Pan02 (HMGB1‐KO) cells, and the BALB/c nude mice were injected with 4 × 10^6^ SW1990 (WT or NLS FBP1) cells, 6 × 10^6^ SW1990 (HMGB1 WT or KO) cells or 3 × 10^6^ SW1990 (HMGB1 WT, HMGB1 KO, HMGB1 KO/HMGB1 or HMGB1 KO/HMGB1 K43/44A mut) cells, for each xenograft in a volume of 150 uL PBS. Xenografts were planted subcutenously in the right flanks, respectively. Tumor volume was calculated using formula volume = (width^2^) × length/2.

### Serum Markers Analysis

To evaluate the liver and kidney functions, the serum levels of GGT, ALT, AST, BUN, and Cr were analyzed by kit according to the manufacturer's protocol. (GGT activity detection kit, Solarbio, BC1220; AST activity detection kit, Solarbio, BC1560; ALT activity detection kit, Solarbio, BC1550; BUN activity detection kit, Solarbio, BC1530; Cr activity detection kit, LEAGENE, TC1193)

### F1,6P‐CarboxyLink Gel Pulldown Assay

The CarboxyLink Gel (Thermo Scientific, 20266) slurry was mixed by end‐over‐end rotation to achieve a uniform gel suspension. An appropriate volume of carboxyLink gel was transferred to a microcentrifuge tube. The tube was centrifuged for 2 min at low speed (1000 × *g*), then the supernatant was carefully removed and discarded. The gel was washed 3–5 times with 2 gel volumes of ultrapure water, centrifuging and removing the supernatant each time. F1,6P was dissolved in 0.1 m imidazole (pH 6) to obtain a 50 mm solution. An equal volume of the 1,6‐Fructose‐bisphosphate solution (D‐fructose 1,6‐diphosphate sodium, Macklin, D832357) was added to the gel and it was mixed well. 1 mg of EDC was weighed and dissolved in 67 uL of 0.1 m imidazole (pH 6). For each microliter of gel used, 2 µL of the EDC solution was added. The gel reaction was mixed by shaking or rotating for 3 h at room temperature. The tube was centrifuged and the supernatant was removed, which contained the non‐bound F1,6P. The gel was washed 3–5 times with 2 gel volumes of water, centrifuging and discarding the supernatant each time. HEK293T cell pellets were washed once with PBS and resuspended in PBS supplemented with protease inhibitor cocktail and 1% Triton X‐100. The cells were then further disrupted by repeated freeze‐thaw cycles and vortex with glass beads. After cell disruption, the lysates were spun down by centrifugation (10 000 × *g* at 4 °C for 10 min). The cell extracts or protein (with PBS or F1,6P) were incubated with CarboxyLink Gel (original or F1,6P) at 4 °C for 2 h. And then, the resin was washed with PBS at 4 °C for three times. Bound proteins were eluted using 1× loading dye per test at 100 °C for 10 min.

### LC‐MS/MS Analysis

LC‐MS/MS analysis and data processing were performed at the Wayen Biotechnologies (Shanghai), China. Briefly, eluted proteins were reduced, alkylated and trypsin digested overnight. Trypsin digestion was stopped by addition of mix solution (ddH2O: acetonitrile: formic acid = 97.9%: 2%: 0.1%) and the peptides were desalted on C18 Cartridges, concentrated by vacuum centrifugation, and reconstituted in 0.1% (v/v) formic acid. LC‐MS/MS analysis was performed on a Bruker maxis impact UHR Q‐TOF (Germany, Bruker). The data were analyzed using Data Analysis (Germany, Bruker). Data were searched against the PEAKS 8.5 database (Canada, BIS company).

### ITC

ITC experiments were carried out using a Malvern‐PEAQ apparatus. Typical titration experiment consisted of 19 or 13 consecutive injections at 150 s intervals into the titration cell. The F1,6P, peptide, and protein (HMGB1∖HMGB1‐A box) were dissolved in the same buffer (100 mm Hepes, 50 mm NaCl) and degassed for 10 min under vacuum in each experiment. Controls included F1,6P injected into buffer, peptide injected into buffer and F1,6P‐peptide injected into buffer solutions, respectively. Data were analyzed by using nonlinear regression with a single site binding model in Malvern MicroCal PEAQ ITC Analysis.

### SPR

The affinity between HMGB1 protein or DNA was determined with a Biacore T200 (cytiva) at 25 °C, in running buffer (100 mm Hepes, 50 mm NaCl). Each aptamer (HMGB1 protein or DNA) was immobilized on a Sensor chip CM5 or SA (Cytiva, 29104988; Cytiva, 29104992), and the interaction of the HMGB1 with HMGB1 or HMGB1 with DNA was detected by monitoring injections of gradient HMGB1 protein (diluted with running buffer) in the Kinetic Injection mode. Measurement conditions were 30 uL min^−1^ flow rate, 120 s/240 s for protein injection time, and 200 s/420 s for dissociation monitoring time. After each injection, the sensor surface was regenerated with a 50 µL injection of 5 mm NaOH or 0.5% SDS, and the aptamer refolding was subsequently accomplished by equilibration with the running buffer for 10 min. To determine the KD values, sensorgrams of both a reference cell (no aptamer immobilization) and a measurement with the running buffer injection were subtracted from each sensorgram of the aptamers. The data were fitted with a 1:1 binding model, using the BIA evaluation T200 software. The 50% of inhibition was calculated by the following equation: IC50 = log^‐1^ [Xm‐i(∑p‐0.5)]

### Particle Size Distribution

The purified HMGB1 protein incubated with or without 200 µm F1,6P at 4 °C for 4 h was detected by Zeta potential and nanoparticle size analyzer(DelsaNano)

### Electron Microscopy

The protein samples treated with or without 0.2 mm F1,6P were fixed by 1% glutaraldehyde at 4 °C for 16 h. They were diluted tenfold in 5 mm triethanolamine/HCl, 0.2 mm EDTA, pH 7. Preparations were observed with Thermo Scientific Talos F200S electron microscope.

### Immunoprecipitation and Pull Down Assay

Protein or cell lysates were incubated with 1 µg of the IgG or antibody and shaken slowly overnight at 4 °C, then 20 µL Protein A+G Agarose (Beyotime, P2055) was added and shaken slowly at 4 °C for 1–3 h. The agarose was then rinsed with PBS for three times and subjected to SDSPAGE. Protein bands were detected by Western blot. Cell pellets were washed once with PBS and resuspended in PBS buffer supplemented with protease inhibitor cocktail and 1% Triton X‐100. The cells were then further disrupted by repeated freeze‐thaw cycles and vortex with glass beads. After cell disruption, the lysates were spun down by centrifugation (12 000 × *g* at 4 °C for 10 min). Protein concentration of whole cell extracts was determined by Bradford assay. The whole cell extracts were incubated with anti‐Flag M2 magnetic beads (Sigma Aldrich, M8823) or HA agarose (Sigma Aldrich, A2095) at 4 °C for 2 h on a rotating wheel. After incubation, the beads or agarose was washed with PBS at 4 °C for three times. Bound proteins were eluted using either 3× Flag peptide in PBS at 4 °C for 30 min or 1× loading buffer at 100 °C for 10 min(heating beads or agarose). Protein bands were detected by Western blot. The purified HMGB1 protein was treated with F1,6P or G6P. The samples were incubated with cisplatin‐treated biotin‐DNA at 4 °C for 1 h. Then, each sample was mixed with 10 µL streptavidin beads (New England Biolabs, S1420S) 4 °C for 1 h. After incubation, the beads were washed with PBS at 4 °C for three times and subjected to SDSPAGE. Protein bands were detected by Western blot.

### Protein Stability Assay

SW1990 cells were pretreated with or without 5 m F1,6P for 24 h, then media were supplemented with 50 ng µL^−1^ cycloheximide (CHX, MedChemExpress, HY‐12320). The cells were harvested at time points 0 h, 1 h, 2 h, 4 h. The levels of TP53 protein were measured using Western Blot.

### Live Cell Imaging System

For GFP expression experiments, the HEK293 cells transfected with VC155‐HMGB1(AB‐box)‐linker‐(C‐tail)‐VN173 or VC155‐HMGB1(AB‐box‐K43AK44A)‐linker‐(C‐tail)‐VN173 plasmid were seeded into 12‐well plates. After treatment, the fluorescence signals were detected by BioTek CYTATION imaging reader.

### Protein Cross‐Linking

Crosslinking assays to detect HMGB1 self‐association, the HMGB1 protein was pre‐incubated with PBS or F1,6P (0.5 mm in PBS) for 30 min at 4 °C, followed by addition of 100 µm BS3 solution (BS3 crosslinker, Biovision, 2327) and incubated at 37 °C for 10 min. Crosslinking was stopped by 20 mm Tris (pH7.5) on ice. Solution after reaction was subjected to SDSPAGE in 10% gels and immunoblotting.

### RNA Extraction and qRT‐PCR Assays

Total RNA was extracted from cultured cells using TRIZOL reagent (Invitrogen, 15596026). For qRT‐PCR, RNA was reverse transcribed to cDNA by using a Reverse Transcription Kit (YEASEN, 11139ES60). Real‐time PCR analyses were performed with Hieff qPCR SYBR Green Master Mix (YEASEN, 11200ES03). Results were normalized to the expression of 18s or beta‐actin. The sequence of the primers was described in “KEY RESOURCES TABLE.” The qRT‐PCR assays were conducted on BioRad CFX96 Real‐Time PCR detection system, and data were collected with this instrument.

### Western Blot Analysis

Cells were collected and lysed in RIPA buffer with 1 mm PMSF and protease inhibitor cocktail. All protein samples were fractionated on SDS‐PAGE and transferred to a PVDF membrane. Immunoblotting analyses were performed with the indicated antibodies overnight at 4 °C with gentle shaking and visualized with horseradish peroxidase‐conjugated secondary antibodies for 1 h at room temperature using a chemiluminescence detection system (Tanon 5200 chemiluminescence imaging system).

### Immunofluorescence

Cells grown on glass bottom plates were washed and fixed with 2.5% paraformaldehyde at room temperature for 15 min. The cells were then treated with blocking and permeabilization buffer (3% BSA and 0.2% Triton X‐100 in PBS) for 1 h at 37 °C. After blocking and permeabilization, the cells were incubated with primary antibodies in antibody dilution buffer (1% BSA and 0.02% Triton x‐100 in PBS) overnight at 4 °C, followed by incubation with appropriate fluorophore‐labeled secondary antibodies in the same buffer for 2 h at 4 °C and then for 5 min with 1 µg mL^−1^ DAPI at room temperature. All immunofluorescence images were taken with the Zeiss 710 LSM confocal microscope.

### DNA Replication Assay

The cells (SW1990 HMGB1 WT & HMGB1 KO) were seeded in 6‐well plates and treated according to grouping conditions. Cells were performed with BeyoClick EdU‐647 Kit (Beyotime, C0081S) according to the manufacturer's protocol. The cells were incubated with 10 µm EdU for 2 h at 37 °C and then postfixed with 4% PFA for 10 min and permeabilized with 0.3% Triton X‐100 in PBS for 15 min, before being incubated with freshly prepared Click‐iT EdU detection cocktail for 30 min at 37 °C. Then incubated with 5 µg mL^−1^ of DAPI to stain the cell nuclei for 10 min. Images were captured using confocal microscopy.

### Fluorescence Imaging after Photobleaching

Hmgb1‐GFP fluorescence was imaged using Zeiss 710 LSM confocal microscope. GFP was excited at 488 nm and their emission was collected between 510 and 550 nm. For time series measurements, cells were irradiated briefly to allow image focusing and minimize photobleaching. An initial image was recorded to select a region of interest before a series of images was collected automatically. Fluorescence was quantified by using Zeiss∖Zen2 software. The Zeiss∖Zen2 application was used for photobleaching and fluorescence recovery measurements with the following settings: 1 pre‐bleach scan, 1 bleaching scan, 15 post‐bleach scans every 5 s.

### NHEJ Analysis

NHEJ‐GFP U2OS cells were transfected with control or HMGB1 siRNA (with or without F1,6P treated) in the absence or presence of I‐SceI expressin. The final GFP positive cells were detected by flow cytometry.

### Comet Assay

Analysis of baseline and cisplatin∖hydroxurea‐induced DNA damage was performed on freshly cells using an alkaline comet assay. Briefly, microscope slides were precoated with a smear of 1% low melting‐point agarose (Sigma Aldrich, A9045) and allowed to dry for 10 min at 37 °C in the dark. Then, combine the cell suspension with 1% agarose (US EVERBRIGHT, A2015) at a ratio 1:10 (v/v) and the gel was cast over the first low melting‐point agarose layer. A tray containing 500 mL of ice‐cold lysis buffer was placed at 4 °C and the slides were gently lowered into it. After cell lysis, slides were placed in a horizontal gel electrophoresis unit filled with alkaline electrophoresis buffer for 1 h at 4 °C. Electrophoresis was then performed at 1 V cm^−1^ for 30 min. Next, the slides were removed and rinsed by placing them in H_2_O for 5 min in twice. Gently immerse slides in 70% ethanol for 5 min at room temperature. SYBR Green dye was added to each slide to stain the DNA. The level of DNA damage was expressed as % DNA in tail. Data were analyzed using CometScore software.

### Cancer Cells with Chemotherapy Resistance Generation and Analysis

U2OS, H209, H69, Hep3B, and H1299 cells were treated with different concentrations of cisplatin (Solarbio, D8810) and etoposide (Solarbio, IE270). Subsequently, the cells were cultured for 1 month at a concentration with a lower apoptotic rate (Cisplatin: H69 2.5 µm, H209 4 µm, U2OS 7 µm, Hep3B 3 µm, H1299 3.5 µm; Etoposide: H69 3 µm, H209 5 µm, U2OS 10 µm, Hep3B 15 µm, H1299 15 µm). The drug‐resistant cells were divided into two groups and treated with the same gradient concentration of the drug as before, one group was treated with 0.5 mm F1,6P at the same time. MTT assay was used to detect cell viability and calculate IC_50_.

### Isolation of Pure, Intact Nuclei via Biochemical Fractionation

The cell pellet was resuspended in five pellet volumes of extraction buffer A (cytoplasmic extraction buffer: Combine 20 mm Tris, pH 7.6, 0.1 mm EDTA, 2 mm MgCl2, 0.5 mm NaF, 0.5 m Na3VO4. To yield ready‐to‐use extraction buffer, protease inhibitors were supplemented by adding 10 µL of 100× protease inhibitor and 10 µL of 100 mm PMSF to 980 µL of extraction buffer A shortly before use. The cells were incubated for 15 min on ice to induce hypotonic swelling of cells as a preparative step for subsequent cell lysis. Nonidet P‐40 was added to obtain a final concentration of 1% and was mixed gently by vortexing or inverting the tube. This induced cell membrane disruption and the release of cytoplasmic proteins while keeping nuclear membranes intact. Broken cells were homogenized by gently pipetting up and down three times.4 °C, 500 × *g* for 5 min. ≈80% of the supernatant was aspirated and transferred it to a new 1.5‐mL microcentrifuge tube (cytoplasmic extract). The residual 20% was thoroughly discarded, 10–15 pellet volumes buffer (as above) was added to the pellet of crude nuclei. The nuclei were gently resuspended by pipetting up and down and further purifying the crude nuclear preparation. The nuclei were pelleted by centrifugation at 4 °C and 500 × *g* for 3 min and the supernatant was roughly discarded. The rest was the nuclear fraction.

### Detect the Concentration of F16P by Using LC/MS‐MS Assay

Prior to analysis, cells were treated under indicated conditions in DMEM media containing 10% FBS and 4.5 g L^−1^ glucose. After treatment, the cells were washed twice with PBS and detached with trypsin‐EDTA, followed by centrifugation for 3 min at 500 × *g*. The supernatant was discarded (for the detection of F1,6P in both nuclear and cytoplasmic components, the cells were collected and nuclear‐cytoplasmic separation was performed), and a 2:2:1 mixture of methanol: acetonitrile: water was added to the cell pellet to extract metabolites. The cells were vortexed, sonicated, and spun to remove the protein pellet. The isolated cell extracts were filtered through a 0.22 µm nylon membrane before LC/MS‐MS analysis. The extracted metabolites were separated using a Waters Acquity UPLC system equipped with an HSS T3 column (1.7 µm, 2.1 × 100 mm). The two mobile phase solutions were 2 mm DBAA, 95:5 water: acetonitrile, pH = 9 (Solution A), and 6 mm DBAA, 15:85 water: acetonitrile (solution B). The chromatographic gradient method was as follows:0–2.5 min, 2% B; 2.5–12 min, 2–20% B; 12–13 min, 20% B; 13–14 min, 20–2% B; and 14–15 min, 2% B. Mass spectrometry analysis was performed with a 5500 QTRAP hybrid triple quadrupole linear ion trap mass spectrometer (AB Sciex, Foster City, CA) equipped with a turbo ion spray electrospray ionization source. All spectra were obtained in negative ion mode. The MS/MS transition 339/79 was monitored under 8 V collision energy for quantification of fructose‐1,6‐bisphosphate. For data analysis, calibration curves of fructose‐1,6‐bisphosphate were constructed using the area under peak versus the concentrations of standards. The concentrations of fructose‐1,6‐bisphosphate in cell extract were determined from the fitted calibration curve. The intracellular concentration of the metabolite was estimated assuming a cell volume of 3.4 cm^3^/10^9^ cells. (Meth. Cell Biol, 1973,7, 361; Adv. Enz. Regul. 1985,23, 81)

### Human Tumor Organoids Generation and Analysis

The generation and analysis of human colon cancer organoids were performed by K2 ONCOLOGY Technology Co., Ltd in a double blind strategy. Patient samples were obtained from Yixing People's Hospital. The ethics was approved by the Ethics Committee of Yixing People's Hospital. Ethics review document batch number: 2020 K 60. Briefly, colon cancer organoids were established and cultured with complete medium in 24‐well plate for 1 week. The organoids were then seeded in 96‐well and treated with different concentrations of F1,6P. The viability of organoids was detected by a luciferase‐based assay.

### Statistical Analysis

Data were presented as the mean ± SD for the indicated number of independent experiments. Significance between indicated groups was determined using the Student's *t*‐test (unpaired two‐tailed, unequal variance); *P < 0.05, **P < 0.01, and ***P < 0.001 were considered significant. The statistical analysis was performed in GraphPad Prism 7 or Excel.

## Conflict of Interest

The authors declare no conflict of interest.

## Supporting information

Supporting Information

Supporting Information

## Data Availability

The data that support the findings of this study are available from the corresponding author upon reasonable request.
