# Supplementary material for: Nuclear Fructose‐1,6‐Bisphosphate Inhibits Tumor Growth and Sensitizes Chemotherapy by Targeting HMGB1
Source: Adv Sci (Weinh). 2023 Jan 15;10(7):2203528. doi: 10.1002/advs.202203528 (PMC9982576; doi:10.1002/advs.202203528)
Supplement: Supplementary file 1 — Supporting Information [file ADVS-10-2203528-s001.pdf]

ADVANCED SCIENCE
Open Access

Supporting Information

for *Adv. Sci.*, DOI 10.1002/advs.202203528

Nuclear Fructose-1,6-Bisphosphate Inhibits Tumor Growth and Sensitizes Chemotherapy by Targeting HMGB1

*Yeyi Li, Yuan Fu, Yan Zhang, Bilian Duan, Yanli Zhao, Man Shang, Ying Cheng, Kai Zhang, Qiujing Yu and Ting Wang**

Supplemental information

Supplemental Information includes one key source table, seven supplemental figure legends and figures.

KEY RESOURCES TABLE

REAGENT or RESOURCE	SOURCE	IDENTIFIER
**Antibodies**		
GFP (B-2) Antibody	Santa Cruz Biotechnology	sc-9996 RRID:AB_627695
GST Tag Polyclonal Antibody	proteintech	10000-0-AP
VeriBlot for IP Detection Reagent (HRP)	Abcam	Ab131366 RRID:AB_2892718
His-tag Antibody	Beyotime	AH367
DYKDDDDK Tag Rabbit PolyAb Antibody	proteintech	20543-1-AP
Flag Antibody	Invitrogen	PA1-984B
Anti-mouse IgG HRP-linked Antibody	cell signaling Technology	7076S RRID:AB_330924
Anti-rabbit IgG HRP-linked Antibody	cell signaling Technology	7074S RRID:AB_2099233
P53 (DO-1) sc-126 mouse monoclonal IgG Antibody	Santa Cruz Biotechnology	sc-126 RRID:AB_628082
Anti-TP53 rabbit polyclonal Antibody	Sangon Biotech	D120082
Ub (P4D1) sc-8017 mouse monoclonal IgG Antibody	Santa Cruz Biotechnology	sc-8017 RRID:AB_2762364
Rb pAb to HMGB1 Antibody	Abcam	Ab18256 RRID:AB_444360
Monoclonal Anti-β-Actin-Peroxidase Antibody	Sigma-Aldrich	A3854 RRID:AB_262011
Anti-phospho-Histone H2AX (ser139) clone JBW301 Antibody	EMD Millipore	05-636 RRID:AB_309864
Histone H2A.X Polyclonal Antibody	proteintech	10856-1-AP
Anti-HA-Peroxidase, High Affin Antibody	Roche	12013819001 RRID:AB_390917
anti rabbit- Alexa Fluor 633Antibody	Invitrogen	A21071 RRID:AB_2535732
Anti rabbit-Alexa Fluor	Invitrogen	A11008

488Antibody		RRID:AB_143165
Anti mouse-Alexa Fluor 488Antibody	Invitrogen	A11001 RRID:AB_2534069
MDM2 Antibody (SMP14)	Santa Cruz Biotechnology	sc-965 RRID:AB_627920
GADD45A Rabbit pAb Antibody	Abclonal	A11768
CDKN1A/p21CIP1 Rabbit mAb Antibody	Abclonal	A19094
ALDOA Polyclonal Antibody	proteintech	11217-1-AP
**Chemicals and Reagents**		
D-fructose 1,6-diphosphate sodium	Macklin	D832357
D-glucose-6-phosphate disodium salt	Shanghai yuanye Bio-Technology	S11024
NADH (disodium salt)	Sangon Biotech	A600642
PBS (phosphate buffered solution)	Solarbio	P1022
FETAL BOVINE SERUM	Gibico	10270-106
RMPI Medium 1640 basic	Gibico	C11875500BT
DMEM basic	Gibico	C11885500BT
MTT (Thiazolyl blue tetrazolium bromide)	Solarbio	M8180
Crystal violet solution, 0.1%	Solarbio	G1064
DMSO (Dimethyl sulfoxide)	Solarbio	D8371
Paraformaldehyde	Sigma-Aldrich	158127
Albuin Bovine	Solarbio	A8010
DAPI dihydrochloride	Sangon Biotech	A606584
Cisplatin	Solarbio	D8810
Etoposide	Solarbio	IE270
Hydroxyurea	Solarbio	H8420
Low melting-point agarose	Sigma-Aldrich	A9045
GDH/TPI	Shanghai yuanye Bio-Technology	S10202
Aldolase	Sigma-Aldrich	A8811
Agarose	US EVERBRIGHT	A2015
Propidium iodide	Invitrogen	P1304MP
Penicillin-Streptomycin Liquid	Solarbio	P1400

Nonessential Amino Acid Solution	Solarbio	N1250
Puromycin	Solarbio	P8230
Trypan blue	Solarbio	C0040
CarboxyLink Coupling Gel	Thermo Scientific	20266
Coomassie brilliant blue R-250	Solarbio	C8430
40% Page Pre-solution (37.5:1)	Solarbio	A1025
N,N,N',N'-Tetramethylethylenediamine	Aladdin	T105496
2-Mercaptoethanol	J&K Scientific	249096
Cycloheximide	MedChemExpress,	HY-12320
Cell Counting Kit8	NCM Biotech	C6005
TRIzol	Invitrogen	15596026
Lipofectamine 2000 reagent	Invitrogen	11668-019
RNAiMAX Transfection Reagent	Invitrogen	13778150
Annexin V-FITC Cell Apoptosis Analysis Kit	SungeneBiotech	AO2001-02P
Protein A+G agrose (Fast Flow, for IP)	Beyotime	P2055-2
Anti-Flag M2 magnetic beads	Sigma-Aldrich	M8823
Monoclonal Anti-HA-Agarose antibody produced in mouse	Sigma-Aldrich	A2095
BS3 crosslinker	Biovision	2327
Series S Sensor Chip CM5	Cytiva	29104988
Series S Sensor Chip SA	Cytiva	29104992
Talon Metal affinity Resin	TaKaRa(Clontech)	635502
Streptavidin Magnetic beads	New England Biolabs	S1420S
OPTI-MEM	Gibico	31985-070
BeyoClick™ EdU Cell Proliferation Kit	Beyotime	C0081S
SYBR Green I (10000×)	Solarbio	SY1020
Hifair® III 1st Strand cDNA Synthesis Kit	YEASEN	11139ES60
Sybr green qPCR master mix	YEASEN	11200ES03
EDTA-free protease inhibitor cocktail	YEASEN	20123ES10
BsmBI-v2	NEB	R0739
T4 DNA ligase	NEB	M0202

Reactive oxygen detection kit	Beyotime	S0033S
Phusion Hot Start II DNA polymerase	Thermo Scientific	F549L
GGT activity detection kit	Solarbio	BC1220
AST activity detection kit	Solarbio	BC1560
ALT activity detection kit	Solarbio	BC1550
BUN activity detection kit	Solarbio	BC1530
Cr activity detection kit	LEAGENE	TC1193
Protein maker	SparkJade	EC0020
**Experimental Models: cell lines**		
SW-1990 cells	ATCC	N/A
Pan02	ATCC	N/A
HepG2	ATCC	N/A
HCT-116	ATCC	N/A
MCF7	ATCC	N/A
NIH3T3	ATCC	N/A
U2OS	ATCC	N/A
H209	Gifted by Dr. Zhe Liu (Tianjin Medical University)	N/A
H69	Gifted by Dr. Zhe Liu (Tianjin Medical University)	N/A
H1299	Gifted by Dr. Tingting Qin (Tianjin Medical University Cancer Institute and Hospital)	N/A
Hep3B	Gifted by Dr. Yueguo Li (Tianjin Medical University Cancer Institute and Hospital)	N/A
U2OS (NHEJ system)	ISceI-U2OS (NHEJ system) were kindly provided by Dr. Lei Shi (Tianjin Medical University)	N/A
**Experimental Models: clinical samples**		
Clinical samples for Human colon tumor organoids	Yixing People's Hospital	N/A

**Experimental Models: mice**		
C57BL/6J mice	Charles River	N/A
BALB/c nude mice	Charles River	N/A
**Oligonucleotides**		
**RNA**	**SOURCE**	**Sequences（5'-3'）**
si HMGB1	Synbio technologies	UGGGUGCUUCUUCUU AUGCdTdT
Si ALDOA	Synbio technologies	1. UUGGAUUUGAUAAC UUGGGdTdT<br>2. UUCUCCUCGGUGUU CUCGGdTdT
Si TP53	Synbio technologies	UCAAAUCAUCCAUUG CUUGdTdT
**Plasmids**	**SOURCE**	**IDENTIFIER**
HA-Ubiquitin	Addgene	#17608
HA-Ubiquitin-K48	Addgene	#17605
HA-Ubiquitin-K48R	Addgene	#17604
pBiFC-VC155	Addgene	#22011
pBiFC-VN173	Addgene	#22010
MDM2-YFP	Addgene	#53962
**Recombinant DNA**	**SOURCE**	**Sequences（5'-3'）**
plenti-HMGB1-Flag	GENEWIZ	Primer 1 F: ATGGGCAAAGGAGATC CTAAGAAGC<br>Primer 1 R: TTCATCATCATCATCTT CTTCTTCATCTTCAT<br>Primer 2 F: CTACCTCGAGCTCAAG CTTCGAATTCGCCACC A TGGGCAAAGGAGATCC TAAGAAGC<br>Primer 2 R: TGTCATCGTCATCCTTG TAGTCGGATCCTTCATC ATCATCATCTTCTTCTT CATCTTCAT
Plenti-NLS-FBP1-Flag	GENEWIZ	Primer F: CTACCTCGAGCTCAAG CTTCGAATTCGCCACC ATGGCTCCAAAGAAGA

		AGCGTAAGGTAATGGCTGACCAGGCGCCC<br>Primer R: TGTCATCGTCATCCTTGTAGTCGGATCCCTGGGCAGAGTGCTTCTCATACA
plenti-HIS-HMGB1	GENEWIZ	Primer F: CCTCGAGCTCAAGCTTCGAATTCGCCACCATGGGCAGCAGCCATCATCATCATCATCAC<br>Primer R: CGCCTCCCCTACCCGGTAGAATTATCTAGACTATTCATCATCATCATCTTCTTCTTCATCTTCAT
plenti-TP53-Flag	GENEWIZ	Primer F: CTACCTCGAGCTCAAGCTTCGAATTCGCCACCATGGAGGAGCCGCAGTCAGATC<br>Primer R: TGTCATCGTCATCCTTGTAGTCGGATCC GTCTGAGTCAGGCCCTTCTGTCTTGAA
pet28a-HIS-TP53	GENEWIZ	Primer 1 F: TGGTGCCTCGTGGTAGCCATATGGAGGAGCCGCAGTCAGATC<br>Primer 1 R: CTCAGCTTCCTTTCGGGCTTTGTTAGTCTGAGTCAGGCCCTTCTGTCTTGAA<br>Primer 2 F: ATGGGCAGCAGCCATCATCATCATCATCACAGCAGCGGCCTGGTGCCTCGTGGTAGCCAT<br>Primer 2 R: TATGCTAGTTATTGCTCAGCGGTGGCAGCAGC

		CAACTCAGCTTCCTTT CGGGCTTTGTTA
pGEX-GST-MDM2-HIS	GENEWIZ	Primer 1 F: GGATCTGGTTCCGCGT GGATCCCCGGAATTCT GCAATACCAACATGTC TGTCTGTACCTACTG Primer 1 R: TCAGTGGTGGTGGTGG TGGTGGGGAAATAAGT TAGCACAATCATTTGA ATTGG Primer 2 F: GGATCTGGTTCCGCGT GGATCCCCGGAATTCT GCAATACCAACATGTC TGTCTGTACCTACTG Primer 2 R: ATGCGGCCGCTCGAGT CGACCCGGGAATTCTC AGTGGTGGTGGTGGTG GTG
plenti-MDM2-HA	GENEWIZ	Primer F: CTACCTCGAGCTCAAG CTTCGAATTCGCCACC ATGTGCAATACCAACA TGTCTGTACCT Primer R: TAATCTGGAACATCGTA TGGGTAGGATCCGGGG AAATAAGTTAGCACAA TCATTTGAAT
plenti-HIS-HMGB1△15	GENEWIZ	Primer F: GAAGATGAAGAGGATG AGGAGGAGGAGTAGT CTAGATAATTCTACCGG GTAGGGGAGGC Primer R: GCCTCCCCTACCCGGT AGAATTATCTAGACTAC TCCTCCTCCTCATCCTC TTCATCTTC
plenti-HMGB1△15-Flag	GENEWIZ	Primer F: CCTCGAGCTCAAGCTT

		CGAATTCGCCACCATG GGCAGCAGCCATCATC ATCATCATCAC<br>Primer R:<br>TGTCATCGTCATCCTTG TAGTCGGATCCCTCCT CCTCCTCATCCTCTTCA TCTTC
Plenti-GFP-HMGB1	GENEWIZ	Primer F:<br>CTCGGCATGGACGAGC TGTACAAGGGATCCAT GGGCAAAGGAGATCCT AAGAAGCC<br>Primer R:<br>CGCCTCCCCTACCCGG TAGAATTATCTAGACTA TTCATCATCATCATCTT CTTCTTCATCTTCAT
plenti-HIS-HMGB1(A-box)	GENEWIZ	Primer F:<br>AGAGAAATGAAAACCT ATATCCCTCCCAAATAG TCTAGATAATTCTACCG GGTAGGGG<br>Primer R:<br>CCCCTACCCGGTAGAA TTATCTAGACTATTTGG GAGGGATATAGGTTTT CATTTCTCT
plenti-HA-HMGB1	GENEWIZ	Primer F:<br>CTACCTCGAGCTCAAG CTTCGAATTCGCCACC ATG<br>TACCCATACGATGTTCC AGATTACGCTATGGGC AAAGGAGATCCTAAGA AGCC<br>Primer R:<br><br>CGCCTCCCCTACCCGG TAGAATTATCTAGACTA TTCATCATCATCATCTT CTTCTTCATCTTCAT
plenti-VC155-HMGB1-VN173	GENEWIZ	Primer 1 F:<br>CTACCTCGAGCTCAAG

		CTTCGAATTCGCCACCatgGACAAGCAGAAGAACGGCATCAAG<br>Primer 1 R: GCTTCTTAGGATCTCCTTTGCCCATGCTGCCGCTGCCCTTGTACAGCTCGTCCATGCC<br>Primer 2F: GGTGGAGGCGGTTCAGGCGGAGGTGGCTCTATGGTGAGCAAGGGCGAGG<br>Primer 2 R: CCCCTACCCGGTAGAATTATCTAGATTACTCGATGTTGTGGCGGATCTTG<br>Primer 3 F: AAGATGAAGATGAAGAAGAAGATGATGATGATGAAGGTGGAGGCGGTTCAGGCGGAGG<br>Primer 3 R: CCCCTACCCGGTAGAATTATCTAGATTACTCGATGTTGTGGCGGATCTTG<br>Primer 4 F: TGAGGAGGAGGAGGAAGATGAAGAAGATGAAGATGAAGAAGAAGATGATGATGATGAA<br>Primer 4 R: CCCCTACCCGGTAGAATTATCTAGATTACTCGATGTTGTGGCGGATCTTG
plenti-HMGB1△C-Flag	GENEWIZ	Primer F: TATCGAGCTAAAGGAAAGCCTGATGCAGCAGGATCCGACTACAAGGATGACGATGACAAG<br>Primer R:

		CTTGTCATCGTCATCCT TGTAGTCGGATCC TGCTGCATCAGGCTTT CCTTTAGCTCGATA
plenti-HIS-HMGB1 A box(K8A)	GENEWIZ	Primer F: GGCAAAGGAGATCCTA AGGCGCCGAGAGGCA AAATGTC Primer R: GACATTTTGCCTCTCG GCGCCTTAGGATCTCC TTTGCC
plenti-HIS-HMGB1 A box(R10A)	GENEWIZ	Primer F: AAGGAGATCCTAAGAA GCCGGCAGGCAAAATG TCATCATATG Primer R: CATATGATGACATTTTG CCTGCCGGCTTCTTAG GATCTCCTT
plenti-HIS-HMGB1 (K43/44A)	GENEWIZ	Primer F: CAGTCAACTTCTCAGA GTTTTCTGCGGCGTGC TCAGAGAGGTGGAAG ACC Primer R: GGTCTTCCACCTCTCT GAGCACGCCGCAGAA AACTCTGAGAAGTTGA CTG
plenti-HIS-HMGB1 A box(K43/44A)	GENEWIZ	Primer F: CAGTCAACTTCTCAGA GTTTTCTGCGGCGTGC TCAGAGAGGTGGAAG ACC Primer R: GGTCTTCCACCTCTCT GAGCACGCCGCAGAA AACTCTGAGAAGTTGA CTG
plenti-VC155-HMGB1(K43/ 44A)-VN173	GENEWIZ	Primer F: CAGTCAACTTCTCAGA GTTTTCTGCGGCGTGC TCAGAGAGGTGGAAG

		ACC<br>Primer R:<br>GGTCTTCCACCTCTCTGAGCACGCCGCAGAAAACTCTGAGAAGTTGACTG
SgHMGB1(Human)-1	GENEWIZ	Primer F:<br>CACCGGCAATATCCTTCCAAAGCAA<br>Primer R:<br>AAACTTGCTTTGGAAGGATATTGCC
SgHMGB1(Human)-2	GENEWIZ	Primer F:<br>CACCGAACAGGCAAGATACTCACGG<br>Primer R:<br>AAACCCGTGAGTATCTTGCCTGTTC
SgHMGB1(Mouse)	GENEWIZ	Primer F:<br>CACCGTCATAAGCTCATACTCACGG<br>Primer R:<br>AAACCCGTGAGTATGAGCTTATGAC
HMGB1-DNA-bingding	GENEWIZ	Primer F:<br>AATCGGAATTCAACCTCTGCCTCCCAA<br>Primer R:<br>TTGGGAGGCAGAGGTTGAATTCCGATT 3'biotin
GADD45A qPCR	GENEWIZ	Primer F:<br>GAGAGCAGAAGACCGAAAGGA<br>Primer R:<br>CACAACACCACGTTATCGGG
P21 qPCR	GENEWIZ	Primer F:<br>TGTCCGTCAGAACCCATGC<br>Primer R:<br>AAAGTCGAAGTTCCATCGCTC
ALDOA qPCR	GENEWIZ	Primer F:<br>ATGCCCTACCAATATCC

		AGCA<br>Primer R: GCTCCCAGTGGACTCATCTG
**Peptides**	**SOURCE**	**Sequences**
HMGB1-C tail-15AA	Synbio technologies	EEDEEDEDEEEDDDE
**Software and Algorithms**		
Excel	Microsoft office	N/A
Adobe Illustrator	Adobe systems	N/A
ImageJ	ImageJ	N/A
BIA evaluation T200	Biacore	N/A
Malvern MicroCal PEAQ ITC Analysis	Malvern	N/A

Supplementary Figure legends

Figure S1. Nuclear accumulation of F1,6P impairs cancer cell viability. a) Indicated cancer cells were stably transformed with control or NLS-FBP1-expressing vectors and analyzed by immunoblotting with indicated antibodies. b) SW1990 cells stably with NLS-FBP1-expressing vector were analyzed by immunofluorescence using anti-Flag antibody and confocal microscopy. Scale bars, 20 μm. c) The nuclear and cytosolic fractions from SW1990 cells stably with NLS-FBP1-expressing vector were analyzed by immunoblotting with indicated antibodies. d) The cell proliferation of indicated cancer cells with indicated F1,6P treatment was analyzed through accounting the cell numbers. e) The tumor colony formation of indicated cancer cells stably with indicated expression vectors was performed and treated with 0.2 mM F1,6P. The colony number was analyzed using crystal violet staining. f) Indicated cancer cells were stably transformed with control or NLS-FBP1-expressing vectors and treated with 5 mM F1,6P. Cell cycle was examined using PI staining and fluorescence-activated cell sorting (FACS) analyses. The ratio of each cell-cycle stage was quantified (See also Figure 1d). g) The cell proliferation of indicated cancer cells with indicated siRNAs were analyzed through accounting the cell numbers (g-1); the ALDOA protein was examined by immunoblotting with indicated antibodies (g-2). h) The relative ROS levels of indicated cancer cells with indicated F1,6P treatment were analyzed by ROS Detection Assay Kit. i) The indicated cells were treated with 5 mM F1,6P, and cell viability was analyzed using a CCK8 assay. j) Cell viability of primary hepatocyte with indicated treatment was analyzed by a MTT assay. k) The indicated cells were treated with 5 mM F1,6P, and the intracellular F1,6P concentration was measured using a F1,6P assay kit. l) The concentrations of F1,6P from indicated fractions of HepG2 cells with 5 mM F1,6P treatment were analyzed by LC-MS/MS. m) A total of 4×10^6 SW1990 cells were subcutaneously injected into athymic nude mice, and F1,6P (10 g/L) was injected intraperitoneally every 2 days. Tumor volume was calculated every 5 days (right), and tumor xenografts on day 30 are shown (left). Data represent the means ± s.d. (n=5 mice per group, **represents $p<0.01$). n-o) Intraperitoneal injection of F1,6P (10 g/L) in C57 mice was performed every 2 days.

The body weight was presented (n), and serum γ -glutamyl transferase (GGT), alanine aminotransferase (ALT), aspartate aminotransferase (AST), urea nitrogen (BUN) and creatinine (GGT), alanine aminotransferase (ALT), aspartate aminotransferase (AST), urea nitrogen (BUN) and creatinine (Cr) were analyzed using Kit (o).

Figure S2. Nuclear F1,6P directly targets HMGB1. a) The HMGB1 protein purified from HEK293 cells was analyzed by Coomassie R-250 Staining. b) The HMGB1-expressed E.coli cell lysate was incubated with F1,6P-resin, and the resin-bounded proteins were eluted by free F1,6P and analyzed by immunoblotting analyses with anti-HMGB1 antibody. c) Indicated cancer cells were treated with 5 mM F1,6P and analyzed by immunoblotting analyses with the indicated antibodies. d) SW1990 cells treated with 5mM F1,6P were analyzed by immunofluorescence with anti-HMGB1 antibody and confocal microscopy. Scale bars, 10 μm. e) Photobleaching analysis of SW1990 cells expressing GFP-HMGB1 treated with 5 mM F1,6P was performed on a confocal microscopy (n= 5). Scale bars, 5 μm. f) The tumor colony formation of SW1990 cells stably with control or HMGB1 sgRNA (clone 2, See also Figure 2f) was performed and treated with 0.2 mM F1,6P. The colony number was analyzed using crystal violet staining.

Figure S3. F1,6P stabilizes p53 protein through HMGB1. a) SW1990 cells treated with 5 mM F1,6P, the immunoblotting analyses were performed using indicated antibodies. b) SW1990 cells with indicated siRNAs treated with 5 mM F1,6P, *GADD45A* and *P21* mRNA levels were analyzed by real-time PCR. c-d) SW1990 cells with the indicated siRNAs (c) and sgRNAs (d) were treated with 5 mM F1,6P, and immunoblotting analyses were performed using the indicated antibodies. e) SW1990 cells with the indicated cycloheximide (CHX) and F1,6P treatment were analyzed by immunoblotting with the indicated antibodies. f) The indicated purified-proteins treated with 0.2 mM F1,6P in the buffer, were subjected to IP with indicated antibodies and further analyzed by immunoblotting with indicated antibodies. g) A Schematic model showing the dynamic association between

HMG-box and C-tail of HMGB1 protein suggested by previous reports. h) The HMGB1 A-box purified from HEK293 cells was analyzed by Coomassie R-250 Staining. i) HEK293 cells expressing GFP(C)-HMGB1-GFP(N) fusion protein with indicated F1,6P treatment were analyzed by immunoblotting with indicated antibodies.

Figure S4. F1,6P breaks up HMGB1 oligomer. a) The SPR of free HMGB1 protein with serially diluted concentrations binding to chip-anchored HMGB1 was analyzed on a Biacore platform. b) A Schematic model showing the effect of F1,6P on HMGB1 self-association.

Figure S5. F1,6P impairs DNA-associated functions of HMGB1. a) SPR of free HMGB1 protein with serially diluted concentrations binding to chip-anchored DNA was analyzed on a Biacore platform. b) The DNA replication efficiency of cells with indicated HU and F1,6P treatment was analyzed by EdU incorporation. Representative images are shown; scale bars, 20 μm. c) SW1990 cells treated with indicated cisplatin (left), HU (right) and F1,6P, the DNA damage was analyzed by comet assay. Representative images are shown; scale bars, 10 μm. d) SW1990 cells with indicated cisplatin and F1,6P treatment were analyzed by immunoblotting analyses with indicated antibodies. e) HMGB1 WT or KO SW1990 cells with indicated F1,6P treatment were analyzed by immunoblotting analyses with indicated antibodies. f) The efficiency of rejoining the I-Scel–induced double-strand DNA breaks was analyzed in U2OScells with indicated treatment by FACS. g) The cell lysates of indicated cells were analyzed by immunoblotting with indicated antibodies. h-i) Indicated HMGB1 WT or KO cells were treated as indicated cisplatin and F1,6P. DNA damage was analyzed by comet assay and representative images (scale bars, 20 μm) are shown (h); cell viability was analyzed using an MTT assay (i).

Figure S6. K43/K44 residues of HMGB1 mediate the anti-tumor effect of F1,6P. a) The HMGB1 structure predicted by Alphafold2 with high confidence. b) The mutated

HMGB1 A-box purified from HEK293 cells was analyzed by Coomassie R-250 Staining. c) The mutated HMGB1 protein purified from HEK293 cells was analyzed by Coomassie R-250 Staining. d) HEK293 cells expressing indicated fusion proteins with indicated F1,6P treatment were analyzed by immunoblotting with indicated antibodies. e) SW1990 cells with the indicated expressing vectors pretreated with 20 uM MG132 were treated with 5 mM F1,6P, and cell extracts were subjected to IP with anti-Flag antibody and analyzed by immunoblotting with the indicated antibodies. f-1) Indicated cancer cells stably with indicated HMGB1 expression were analyzed by immunoblotting analyses with indicated antibodies. f-2) Indicated cancer cells stably transformed with the indicated HMGB1 expression vectors were treated with 5 mM F1,6P, cell proliferation was analyzed by counting the cell numbers.

Figure S7. F1,6P sensitizes chemotherapy. a-b) The apoptosis of HMGB1 WT (a) or KO (b) SW1990 cells with indicated F1,6P and cisplatin treatment were analyzed by Annexin V/PI staining and FACS. c-1) Pano2 cells stably with or without sgHMGB1 were treated as indicated cisplatin and F1,6P. c-1) cell viability was analyzed using an MTT assay; c-2) cell lysates were analyzed by immunoblotting analyses with indicated antibodies. d) Indicated cancer cells were treated with 0.5 mM F1,6P. Cell viability was analyzed using a MTT assay. e) Indicated cancer cells with or without cisplatin or etoposide pretreatment were treated with cisplatin and etoposide as indicated with or without 0.5 mM F1,6P. Cell viability was analyzed using an MTT assay. f) The effect of F1,6P treatment on colon cancer organoid were analyzed through a luciferase-based strategy.

Figure. S1-1

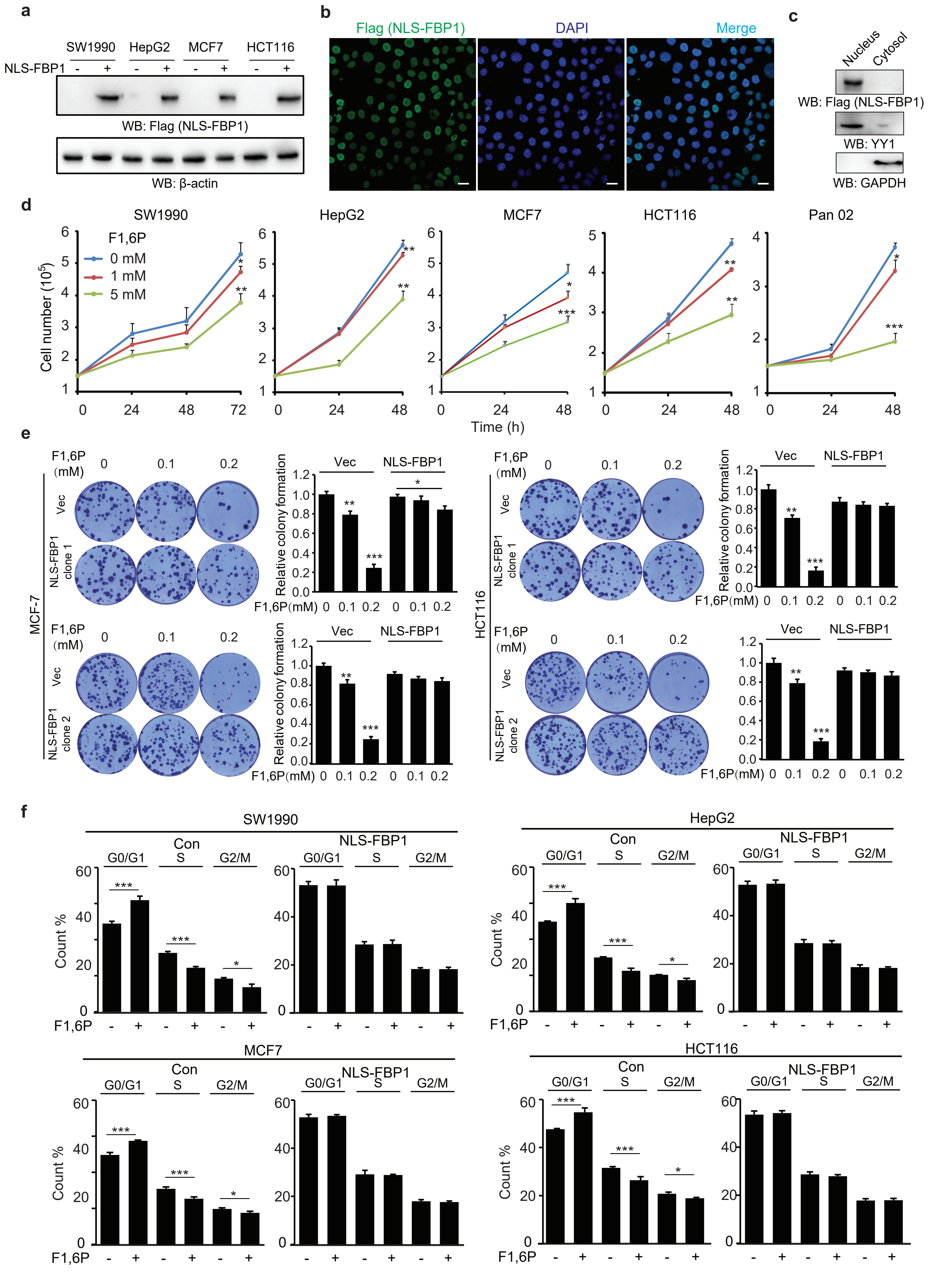

a
SW1990
HepG2
MCF7
HCT116
NLS-FBP1
WB: Flag (NLS-FBP1)
WB: β-actin
b
Flag (NLS-FBP1)
DAPI
Merge
c
Nucleus
Cytosol
WB: Flag (NLS-FBP1)
WB: YY1
WB: GAPDH
d
SW1990
HepG2
MCF7
HCT116
Pan 02
F1,6P
0 mM
1 mM
5 mM
Cell number (10^5)
Time (h)
e
F1,6P (mM)
Vec
NLS-FBP1 clone 1
NLS-FBP1 clone 2
MCF-7
HCT116
Relative colony formation
F1,6P(mM)
f
SW1990
HepG2
MCF7
HCT116
Con
NLS-FBP1
G0/G1
S
G2/M
Count %
F1,6P

Figure. S1-2

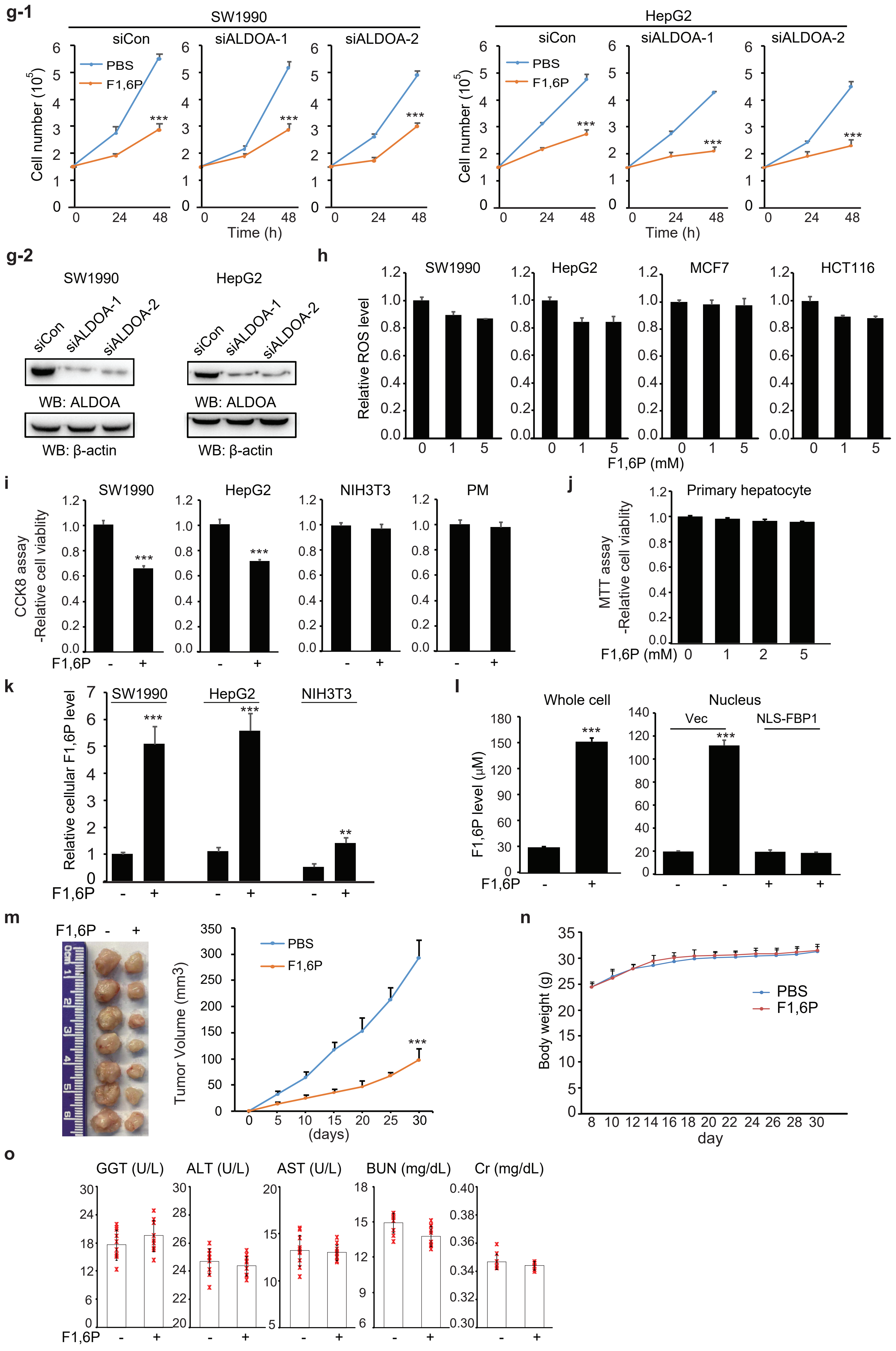

g-1
SW1990
HepG2
siCon
siALDOA-1
siALDOA-2
PBS
F1,6P
Cell number (10^5)
Time (h)
***
g-2
SW1990
HepG2
siCon
siALDOA-1
siALDOA-2
WB: ALDOA
WB: β-actin
h
SW1990
HepG2
MCF7
HCT116
Relative ROS level
F1,6P (mM)
i
SW1990
HepG2
NIH3T3
PM
CCK8 assay
-Relative cell viablity
F1,6P
j
Primary hepatocyte
MTT assay
-Relative cell viablity
F1,6P (mM)
k
SW1990
HepG2
NIH3T3
Relative cellular F1,6P level
F1,6P
l
Whole cell
Nucleus
Vec
NLS-FBP1
F1,6P level (μM)
F1,6P
m
F1,6P - +
PBS
F1,6P
Tumor Volume (mm^3)
(days)
n
PBS
F1,6P
Body weight (g)
day
o
GGT (U/L)
ALT (U/L)
AST (U/L)
BUN (mg/dL)
Cr (mg/dL)
F1,6P

Figure. S2

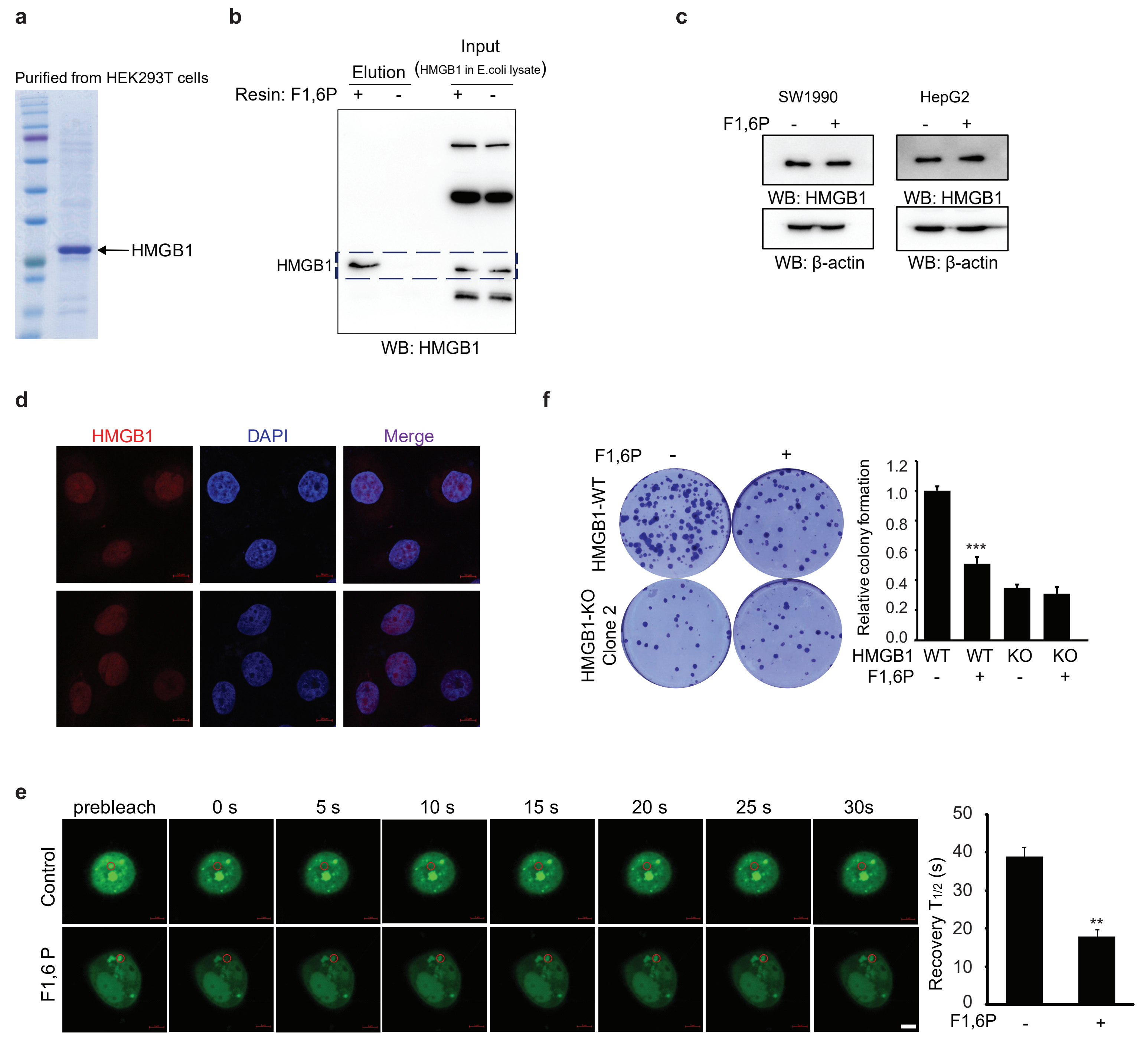
a
Purified from HEK293T cells
HMGB1
b
Input
Elution
(HMGB1 in E.coli lysate)
Resin: F1,6P + - + -
HMGB1
WB: HMGB1
c
SW1990
HepG2
F1,6P - + - +
WB: HMGB1
WB: HMGB1
WB: β-actin
WB: β-actin
d
HMGB1
DAPI
Merge
f
F1,6P - +
HMGB1-WT
HMGB1-KO Clone 2
Relative colony formation
1.2
1.0
0.8
0.6
0.4
0.2
0.0
***
HMGB1 WT WT KO KO
F1,6P - + - +
e
prebleach
0 s
5 s
10 s
15 s
20 s
25 s
30s
Control
F1,6 P
Recovery T1/2 (s)
50
40
30
20
10
0
**
F1,6P - +

Figure. S3

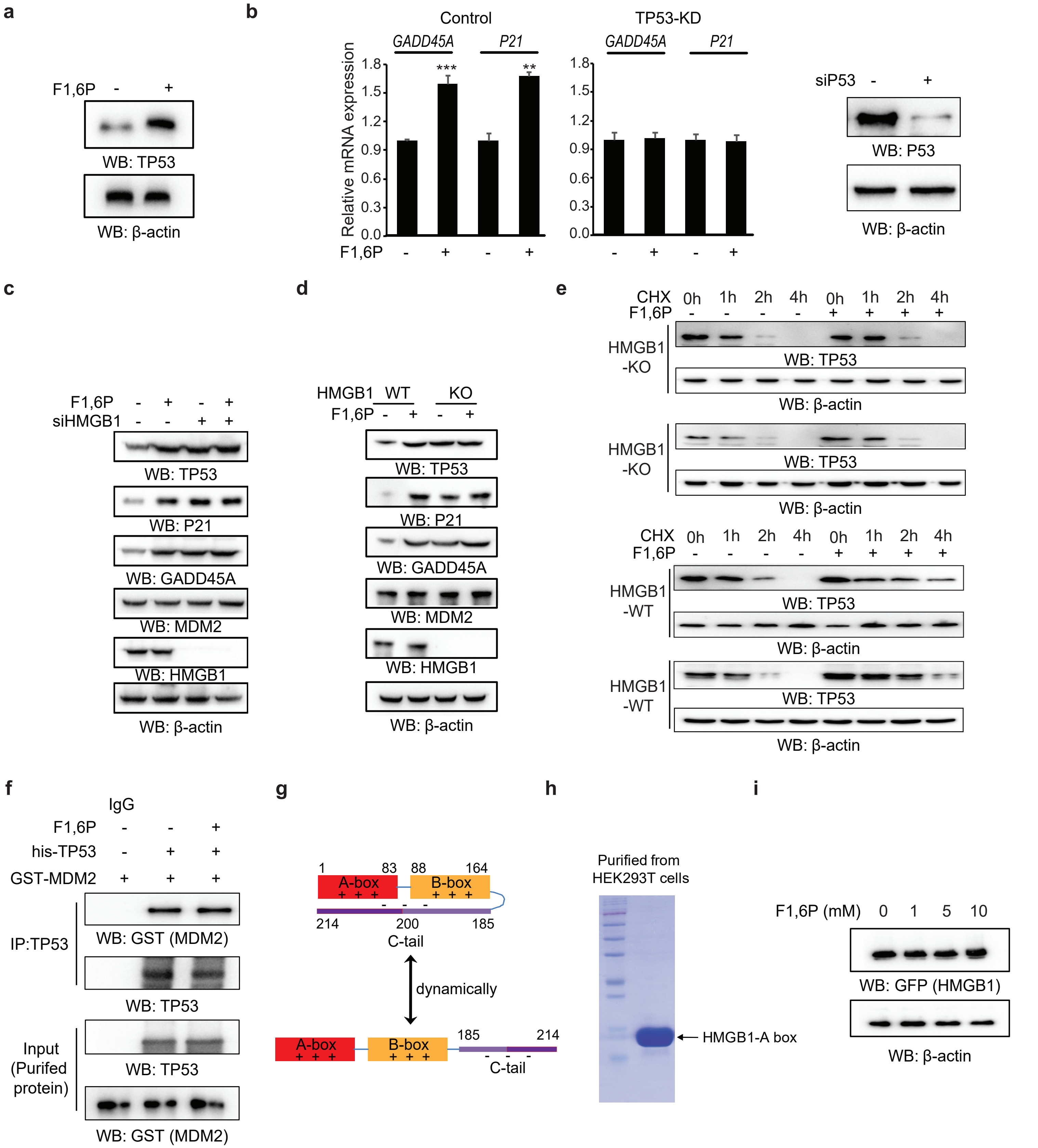
a
F1,6P - +
WB: TP53
WB: β-actin
b
Control
TP53-KD
GADD45A
P21
Relative mRNA expression
1.8
1.5
1.2
0.9
0.6
0.3
0.0
***
**
F1,6P - + - +
siP53 - +
WB: P53
WB: β-actin
c
F1,6P - + - +
siHMGB1 - - + +
WB: TP53
WB: P21
WB: GADD45A
WB: MDM2
WB: HMGB1
WB: β-actin
d
HMGB1 WT KO
F1,6P - + - +
WB: TP53
WB: P21
WB: GADD45A
WB: MDM2
WB: HMGB1
WB: β-actin
e
CHX 0h 1h 2h 4h 0h 1h 2h 4h
F1,6P - - - - + + + +
HMGB1 -KO
WB: TP53
WB: β-actin
HMGB1 -WT
f
IgG
F1,6P - - +
his-TP53 - + +
GST-MDM2 + + +
IP:TP53
WB: GST (MDM2)
WB: TP53
Input (Purifed protein)
g
1 83 88 164
A-box
B-box
214 200 185
C-tail
dynamically
h
Purified from HEK293T cells
HMGB1-A box
i
F1,6P (mM) 0 1 5 10
WB: GFP (HMGB1)

Figure. S4

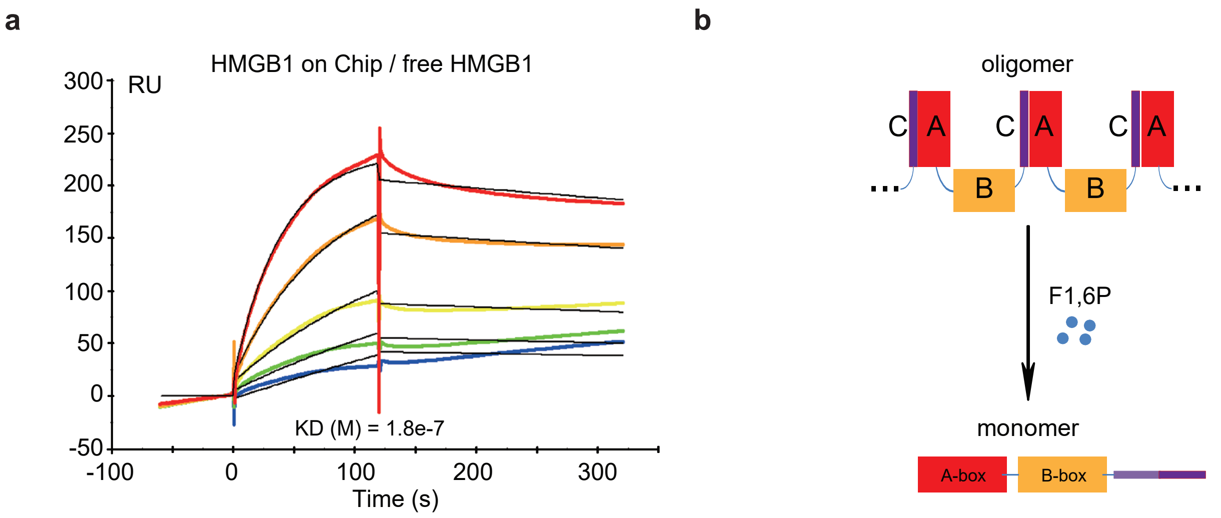
a
HMGB1 on Chip / free HMGB1
RU
300
250
200
150
100
50
0
-50
KD (M) = 1.8e-7
-100
0
100
200
300
Time (s)
b
oligomer
C
A
C
A
C
A
B
B
F1,6P
monomer
A-box
B-box

Figure. S5-1

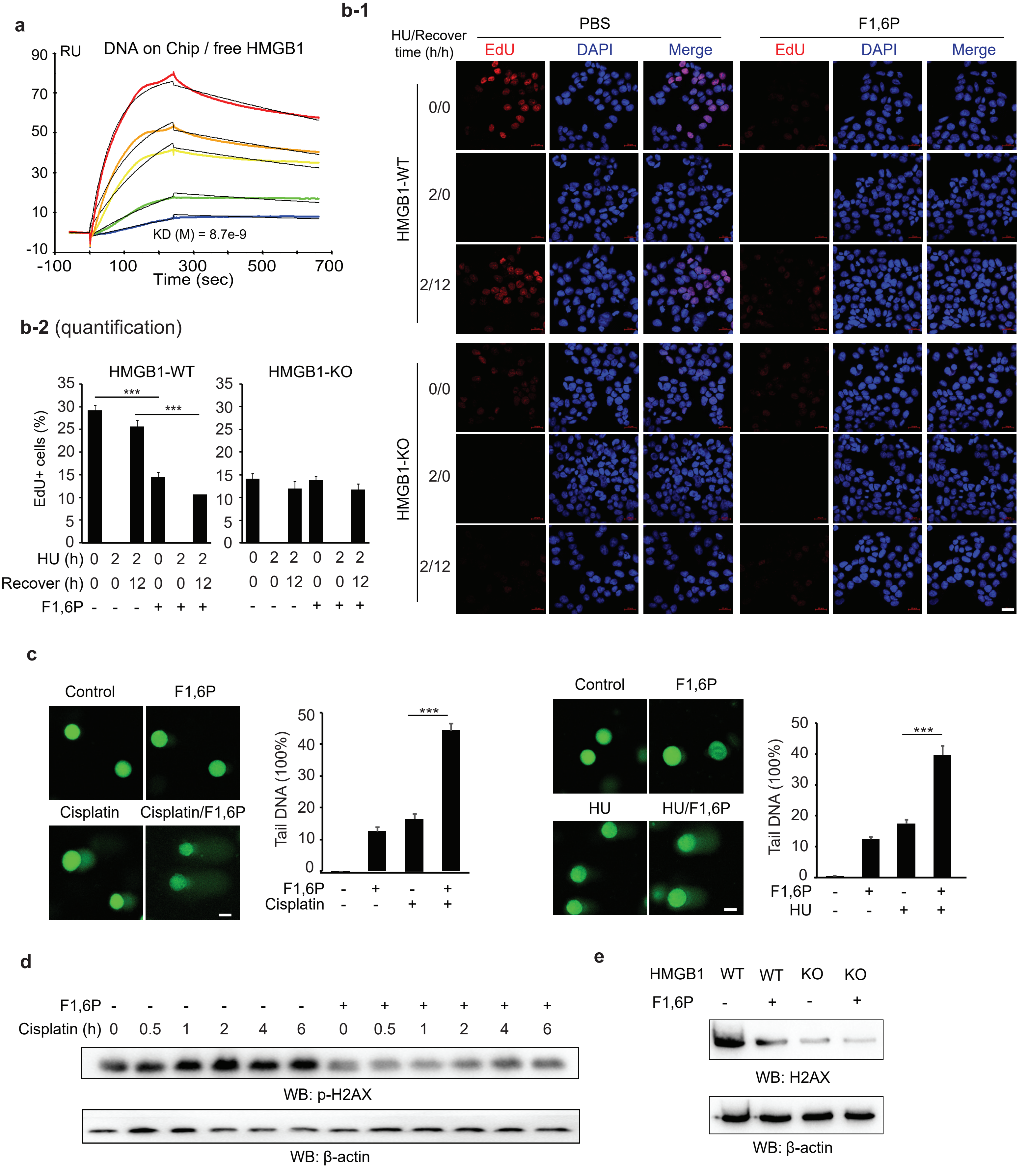
a
RU
DNA on Chip / free HMGB1
KD (M) = 8.7e-9
Time (sec)
b-1
HU/Recover time (h/h)
PBS
F1,6P
EdU
DAPI
Merge
HMGB1-WT
HMGB1-KO
0/0
2/0
2/12
b-2 (quantification)
HMGB1-WT
HMGB1-KO
EdU+ cells (%)
HU (h)
Recover (h)
F1,6P
c
Control
F1,6P
Cisplatin
Cisplatin/F1,6P
Tail DNA (100%)
HU
HU/F1,6P
d
F1,6P
Cisplatin (h)
WB: p-H2AX
WB: β-actin
e
HMGB1
WT
KO
WB: H2AX
WB: β-actin

Figure. S5-2

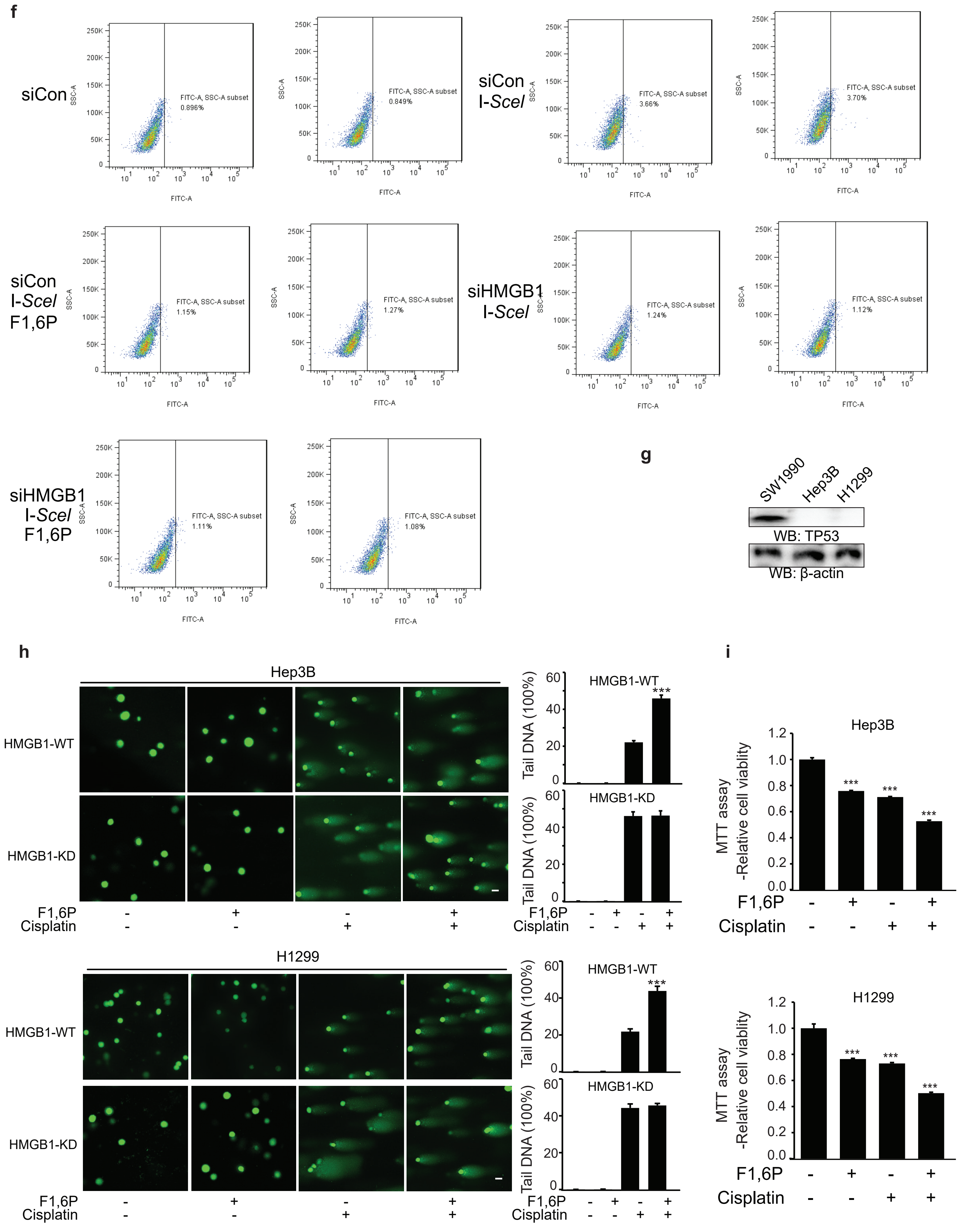

f
siCon
FITC-A, SSC-A subset
0.896%
FITC-A, SSC-A subset
0.849%
siCon
I-*SceI*
FITC-A, SSC-A subset
3.66%
FITC-A, SSC-A subset
3.70%
siCon
I-*SceI*
F1,6P
FITC-A, SSC-A subset
1.15%
FITC-A, SSC-A subset
1.27%
siHMGB1
I-*SceI*
FITC-A, SSC-A subset
1.24%
FITC-A, SSC-A subset
1.12%
siHMGB1
I-*SceI*
F1,6P
FITC-A, SSC-A subset
1.11%
FITC-A, SSC-A subset
1.08%
SSC-A
FITC-A
g
SW1990
Hep3B
H1299
WB: TP53
WB: β-actin
h
Hep3B
HMGB1-WT
HMGB1-KD
F1,6P
Cisplatin
Tail DNA (100%)
H1299
i
Hep3B
H1299
MTT assay
-Relative cell viability
F1,6P
Cisplatin
***

Figure. S6

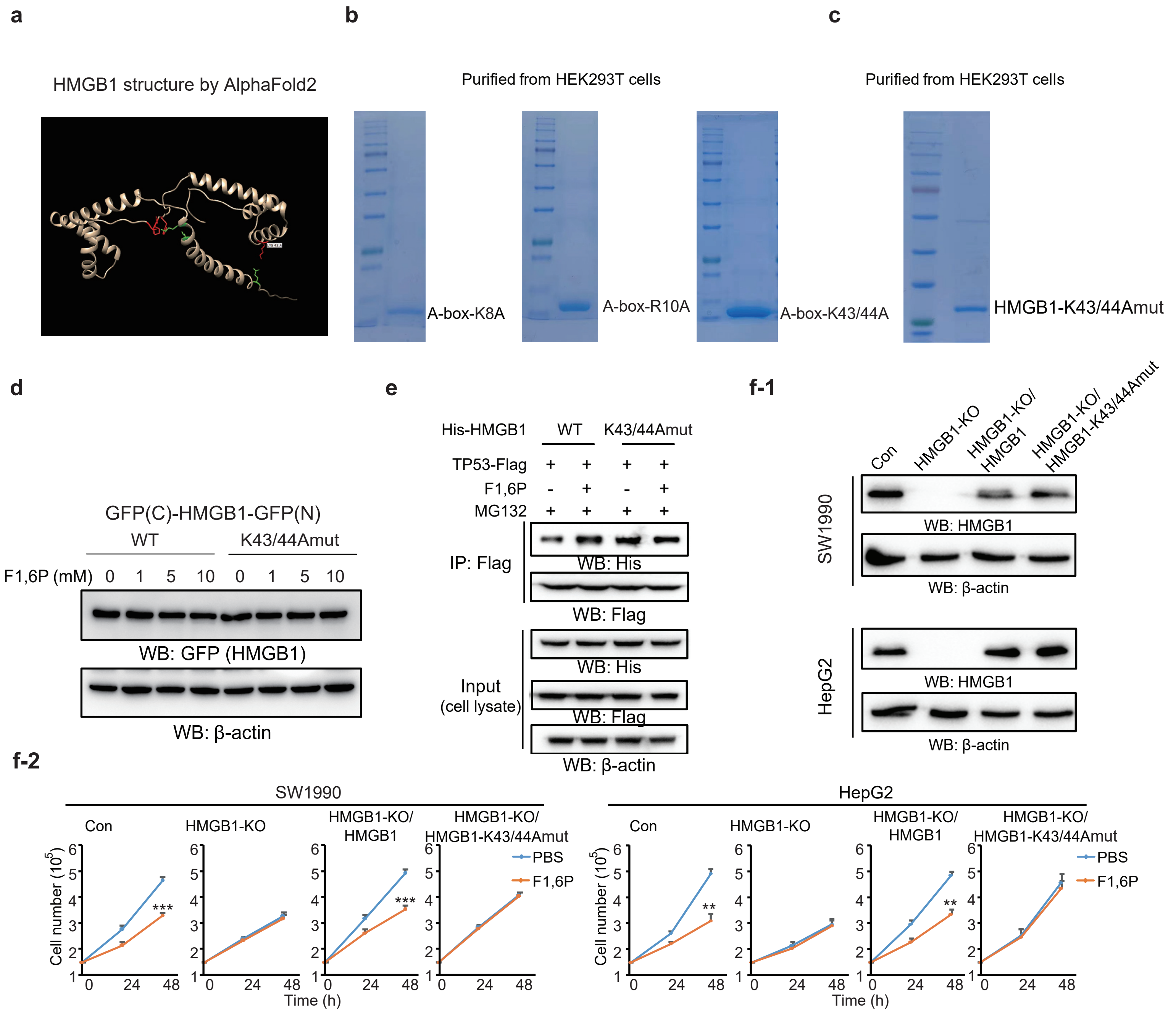
a
HMGB1 structure by AlphaFold2
b
Purified from HEK293T cells
A-box-K8A
A-box-R10A
A-box-K43/44A
c
Purified from HEK293T cells
HMGB1-K43/44Amut
d
GFP(C)-HMGB1-GFP(N)
WT
K43/44Amut
F1,6P (mM) 0 1 5 10 0 1 5 10
WB: GFP (HMGB1)
WB: β-actin
e
His-HMGB1 WT K43/44Amut
TP53-Flag + + + +
F1,6P - + - +
MG132 + + + +
IP: Flag
WB: His
WB: Flag
Input
(cell lysate)
WB: His
WB: Flag
WB: β-actin
f-1
Con
HMGB1-KO
HMGB1-KO/
HMGB1
HMGB1-KO/
HMGB1-K43/44Amut
SW1990
WB: HMGB1
WB: β-actin
HepG2
WB: HMGB1
WB: β-actin
f-2
SW1990
HepG2
Con
HMGB1-KO
HMGB1-KO/
HMGB1
HMGB1-KO/
HMGB1-K43/44Amut
Cell number (10^5)
Time (h)
PBS
F1,6P
***
**

Figure. S7

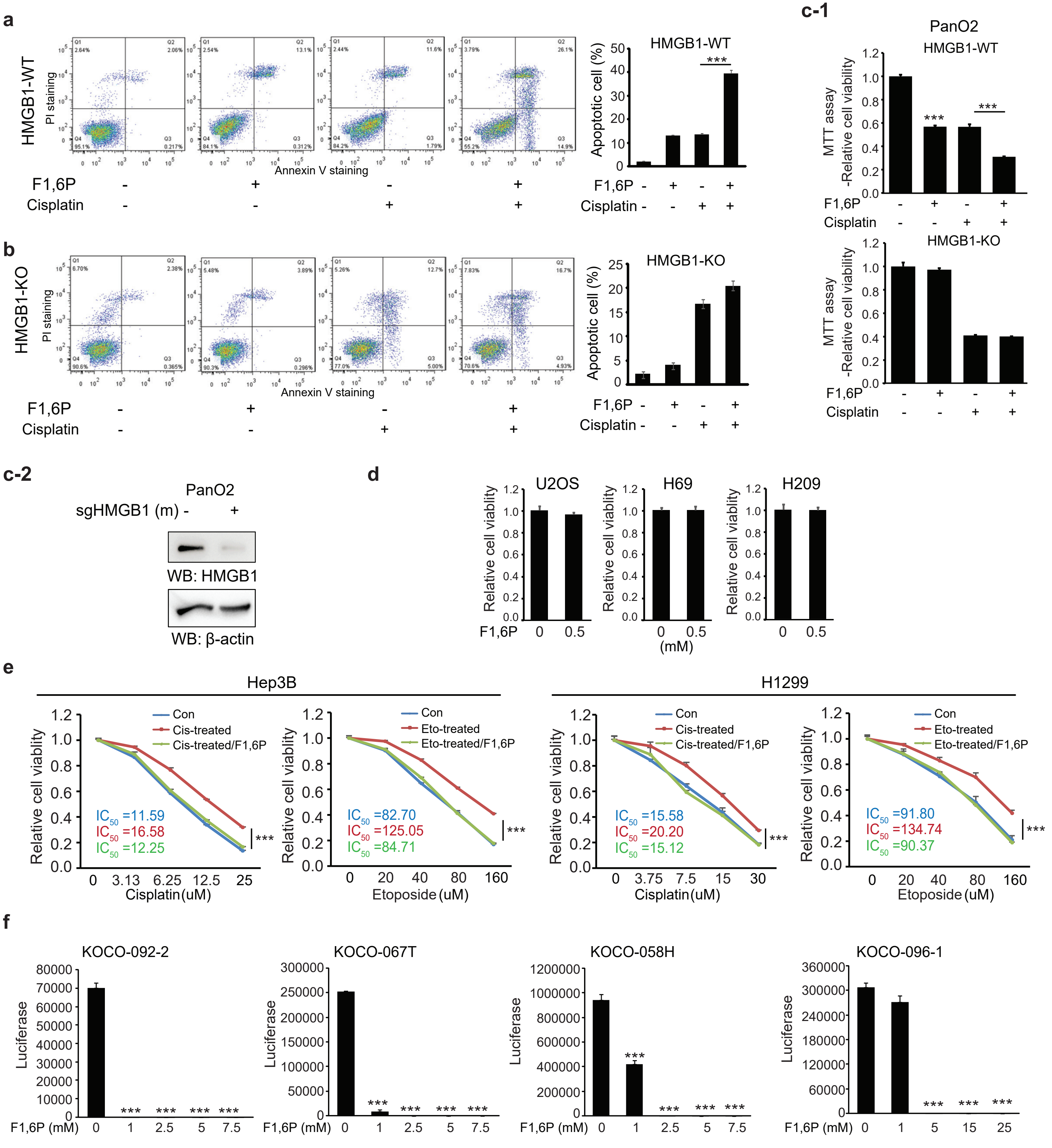

a
HMGB1-WT
PI staining
Annexin V staining
F1,6P - + - +
Cisplatin - - + +
HMGB1-WT
Apoptotic cell (%)
***
c-1
PanO2
HMGB1-WT
MTT assay
-Relative cell viability
***
***
F1,6P - + - +
Cisplatin - - + +
HMGB1-KO
b
HMGB1-KO
PI staining
Annexin V staining
Apoptotic cell (%)
c-2
PanO2
sgHMGB1 (m) - +
WB: HMGB1
WB: β-actin
d
U2OS
H69
H209
Relative cell viability
F1,6P 0 0.5
(mM)
e
Hep3B
H1299
Con
Cis-treated
Cis-treated/F1,6P
Eto-treated
Eto-treated/F1,6P
Relative cell viability
IC50 =11.59
IC50 =16.58
IC50 =12.25
IC50 =82.70
IC50 =125.05
IC50 =84.71
IC50 =15.58
IC50 =20.20
IC50 =15.12
IC50 =91.80
IC50 =134.74
IC50 =90.37
***
Cisplatin (uM)
Etoposide (uM)
f
KOCO-092-2
KOCO-067T
KOCO-058H
KOCO-096-1
Luciferase
***
F1,6P (mM)
